# Interpretable and extrapolation-stable model for predicting nanofluid thermal conductivity

**DOI:** 10.1038/s41598-026-52822-y

**Published:** 2026-05-25

**Authors:** E. Zinhom, S. S. Radwan, A. Elmasry, M. A. M. Sharaf, M. M. Nassar

**Affiliations:** 1 Department of Mathematics, Faculty of Science, Ain Shams University, Cairo, Egypt; 2 Department of Mathematics, Faculty of Science Girls Section, AlAzhar University, Cairo, Egypt

**Keywords:** Nanofluid thermal conductivity, Hybrid modeling, Generalized additive models (GAMs), Residual learning, Outlier detection, Machine learning, Engineering, Mathematics and computing

## Abstract

Accurate prediction of nanofluid thermal conductivity is essential for the design of advanced thermal systems; however, existing approaches often face a trade-off between predictive accuracy and physical interpretability. In this study, a physics-guided hybrid modeling framework is proposed by integrating a Generalized Additive Model (GAM) with a Gradient Boosting Machine (GBM) to address this limitation. The proposed methodology combines a spline-based GAM to capture global thermophysical trends with a regularized boosting model to learn localized nonlinear corrections. A comprehensive preprocessing pipeline is implemented, including feature engineering, Box-Cox transformation for variance stabilization, and a Random Forest-based outlier detection strategy. The hybrid model is evaluated against several state-of-the-art machine learning methods, including Random Forest, Support Vector Regression, Gaussian Processes, Neural Networks, and Decision Trees. The results demonstrate that the proposed framework achieves high predictive accuracy (RMSE $\thickapprox 0.0048$ W/mK) while maintaining physically consistent behavior across the studied domain. The model’s generalization capability is further validated using a Leave-One-Group-Out (LOGO) protocol, where it exhibits stable performance when predicting unseen base fluids. Additionally, the hybrid approach produces smooth and interpretable response profiles that align with known thermodynamic trends. These findings indicate that physics-guided hybrid modeling provides a balanced and reliable approach for thermophysical property prediction, offering improved interpretability without compromising predictive performance.

## Introduction

Nanofluids, colloidal suspensions of nano-sized particles (1–100 nm) dispersed in traditional base fluids such as water, ethylene glycol, or engine oil, have emerged as promising working fluids for advanced thermal management applications. As modern engineering systems (e.g., microelectronics, automotive engines, and solar thermal collectors) continue to experience miniaturization and increased power densities, the thermal limitations of conventional heat transfer fluids have become a critical bottleneck^1^. Incorporating high conductivity metallic or metal-oxide nanoparticles (e.g., $Al_2O_3$, *CuO*, $TiO_2$) has been shown to significantly enhance thermal conductivity, often exceeding predictions from classical mixture theories such as those by^2^ and^3^.

### Thermal conductivity behavior of nanofluids

Extensive experimental research has established that the thermal conductivity ($k_{nf}$) of nanofluids depends on several key factors, including temperature, nanoparticle volume fraction, particle size, particle shape, and base-fluid properties. Studies by^4–6^, and^7^ consistently demonstrate that thermal conductivity increases with rising temperature. Likewise, the nanoparticle volume fraction plays a vital role: investigations by^8^ and^9^ report that higher particle loadings generally yield greater thermal enhancement. The influence of particle size has also been documented; works by^10^ and^11^ indicate that, under certain dispersion conditions, larger nanoparticles may contribute to increased thermal conductivity. These studies underscore the complex multivariate nature of nanofluid heat-transfer behavior.

### Limitations of traditional models

Despite substantial progress, accurate modeling of nanofluid thermal conductivity remains challenging. Experimental measurements are often laborious, expensive, and susceptible to instability, while theoretical correlations frequently fail to capture critical physical mechanisms such as Brownian motion, nanoparticle clustering, interfacial layering, and temperature-dependent effects. As a result, significant discrepancies persist between theoretical predictions and experimental data, motivating the development of more flexible and data-driven modeling approaches.

### Rise of machine learning methods

To address these limitations, machine learning (ML) techniques have gained traction as tools for modeling nanofluid thermal conductivity. Algorithms such as Linear Regression (LR), Support Vector Regression (SVR), Gaussian Process Regression (GPR), and Artificial Neural Networks (ANN) have demonstrated strong predictive performance by uncovering nonlinear and multidimensional relationships within experimental datasets. ANN models, in particular, have been widely adopted; for example,^12,13^, and^14^ applied ANN-based frameworks with notable success. SVR has also shown promise, as demonstrated by^15^, who reported superior accuracy compared to ANN. Although used less frequently, GPR a gray-box model, has been recognized by^16^ as a highly effective regression strategy, offering strong predictive accuracy and natural uncertainty quantification.

In addition to these methods, ensemble learning approaches such as Random Forest (RF) a bagging-based model composed of multiple decision trees, have emerged as powerful black-box predictors. RF models are known for their robustness to noise, ability to capture nonlinear interactions, and strong generalization performance, making them suitable candidates for nanofluid property prediction when interpretability is not the primary objective^17^. To mitigate this opacity, recent studies have begun applying post-hoc explainability techniques; for instance,^18^ utilized Shapley Additive Explanations (SHAP) and Local Interpretable Model-agnostic Explanations (LIME) to decipher the complex transport mechanisms captured by hybrid nanofluid models.

Beyond post-hoc interpretability, recent advancements in computational engineering have increasingly relied on sophisticated, physically grounded architectures that embed physical laws directly into the learning process. For example, Symbolic Regression offers the distinct advantage of distilling complex datasets into explicit, interpretable mathematical formulations. The combination of physical constitutive equations with deep learning architectures to create Physics-Informed Neural Networks (PINNs) is a major breakthrough. These advanced frameworks have been successfully deployed across diverse and highly complex engineering domains, ranging from advanced composite structural analysis and tunnel engineering to broad thermophysical and failure modeling ^19–27^.

While PINNs and Symbolic Regression represent the cutting edge of fully integrated physical solvers, their deployment often requires massive computational overhead and complex partial differential equation (PDE) formulations.

### The dilemma: thermodynamic consistency vs. predictive fidelity

In the domain of thermophysical characterization, model interpretability is not merely a preference but a prerequisite for validation^28^. For critical thermal management systems, engineers must verify that the predicted behavior, such as the nonlinear rise in conductivity with temperature, adheres to established thermodynamic laws. Generalized Additive Models (GAMs)^29^ offer a compelling statistical framework for this purpose, utilizing B-spline basis functions to enforce smooth, physically plausible trendlines that are inherently transparent.

Nevertheless, a fundamental tension persists between thermodynamic consistency and predictive fidelity. While GAMs excel at capturing global physical laws (the “low-frequency” signal), they often suffer from oversmoothing, failing to capture the sharp, local variations and complex interaction effects inherent in heterogeneous nanofluid datasets. Conversely, powerful ensemble methods like Gradient Boosting Machines (GBM) operate as opaque “black boxes”, achieving high precision by memorizing local data patterns at the cost of physical transparency.

### Gap in the literature

Despite increasing adoption of ML in nanofluid research, no prior work has systematically evaluated a hybrid semi-parametric framework that couples mixed-effects statistical modeling with residual learning. Furthermore, existing studies often rely on basic standard deviation filtering for outlier detection, which fails to account for the multivariate nature of nanofluid datasets, leading to either retention of noise or loss of valid experimental signals.

### Scope and contributions of this study

To bridge the gap between statistical transparency and algorithmic precision, this study proposes a novel Physics-Guided Hybrid Framework for predicting the thermal conductivity of nanofluids. We integrate a GAM to capture the physics-based “base signal” (global trends and fluid-specific baselines) and a GBM to learn the complex residuals (local non-linearities) that the base model cannot resolve.

**Table 1:** Comparative Analysis of Modeling Frameworks: Positioning the Proposed Hybrid Approach.

Feature	White-Box (e.g. Pure GAM)	Gray-Box (Proposed) (e.g. Hybrid (GAM + GBM) / GP)	Black-Box (e.g. GBM / MLP)
Core Logic	**Explicit Physics:** Modeled strictly via additive smooth functions (Splines).	**Physics-Guided:** Global physics defined by GAM; local residuals learned by Boosting.	**Implicit Learning:** Complex, entangled interactions learned purely from data.
Equation	$y = \sum f_j(x_j) + \epsilon$	$y = \underbrace{f_{phys}(x)}_{\text {GAM}} + \underbrace{f_{corr}(x)}_{\text {GBM}}$	$y = \sum _{m=1}^M \nu h_m(x)$
Interpretability	**High:** Full visibility of partial dependence shapes.	**Mixed:** Global trends are transparent; residual corrections are opaque.	**Low:** Requires post-hoc tools (SHAP); internal logic is hidden.
Extrapolation (LOGO)	Stable but biased (often underfits complex data).	**Superior:** Physical constraints enable robust transfer to unseen fluids.	**Poor:** High risk of overfitting; fails to generalize outside training bounds.

The principal contributions of this work are as follows:

1. P*hysics-Guided Architecture* We develop a decoupled two-stage architecture where a GAM explicitly models thermodynamic trends (Temperature, Size, Concentration) using cubic B-Splines and Fixed Effects, while a GBM ensemble corrects local deviations via iterative residual learning.
2. *Context-Aware Outlier Detection* We implement a Random Forest-based outlier detection protocol to strictly filter experimental noise ($>2.5\sigma$) prior to training, ensuring the model learns from physical signals rather than artifacts.
3. *Variance Stabilization* We utilize a Box-Cox transformation to rigorously address the inherent heteroscedasticity and positive skewness of thermal conductivity data, stabilizing the variance for more reliable statistical inference.
4. *Comprehensive Benchmarking* We compare this Hybrid approach against seven state-of-the-art baselines (Standard GBM, RF, SVR, GP, MLP Neural Networks, Bagging, and Decision Trees (DT)).

To rigorously define the trade-offs addressed by our framework, we categorize the methodologies into three distinct classes, as summarized in Table 1.

In addition to the above contributions, this study provides methodological advances regarding the handling of categorical experimental data (Base Fluids vs. Nanoparticles) through Fixed Effects modeling. This ensures that the model learns rigorous, fluid-specific physical baselines rather than overfitting to the idiosyncrasies of specific experimental datasets.

### Organization of the paper

The remainder of this manuscript is organized as follows:

**Section **Data and methodology details the data curation process and the mathematical formulation of the proposed Physics-Guided Machine Learning (PGML) architecture. It characterizes the experimental nanofluid dataset, outlines the RF outlier filtration pipeline, and explains the Box-Cox variance stabilization procedure. Furthermore, it explicitly defines the two-stage hybrid framework: establishing a continuous thermodynamic baseline via a physics-informed GAM utilizing B-splines, followed by high-fidelity, data-driven residual correction using a GBM. **Section **Results and discussion presents a comprehensive performance evaluation, physical validation, and scalability analysis of the framework. It first benchmarks the Hybrid model’s interpolative accuracy against industry-standard black-box algorithms (DT, SVR, Bagging Ensembles, RF, GBM, MLP) and gray-box probabilistic models GP. Subsequently, it assesses strict extrapolative capabilities using the Leave-One-Group-Out (LOGO) protocol and quantifies aleatoric noise via 95% Prediction Intervals (PI). Crucially, this section visualizes the learned partial dependence profiles to confirm the model’s thermodynamic consistency, directly benchmarking the ML trends against modern Effective Medium Theories and established empirical correlations. The section concludes with an analysis of computational execution time and inference scalability.

Finally, **Section **Conclusion synthesizes the core findings, defines the physical boundaries governing the model’s extrapolative capacity, and offers actionable recommendations for deploying PGML frameworks in real-world thermal management tasks. It also outlines promising future research directions, including the transition toward PINNs and surrogate physical solvers.

## Data and methodology

**Fig. 1:**
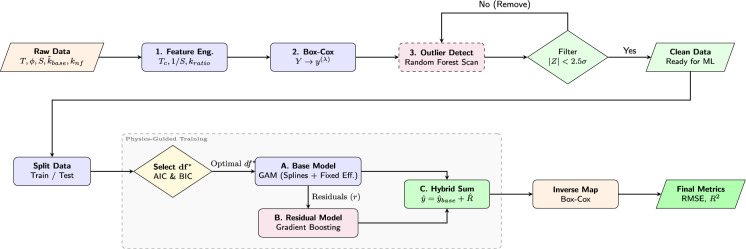
Methodology Flowchart: A horizontal progression from rigorous data cleaning to the hybrid GAM+Boosting training framework.

The computational framework proposed in this study follows a rigorous PGML paradigm, as illustrated in Fig. 1. Following a comprehensive preliminary phase of physics-informed feature engineering, variance stabilization, and outlier filtration, the core predictive architecture is executed across three sequential stages: (1) establishing a continuous, global thermodynamic baseline using a physics-informed GAM, (2) isolating and correcting localized, high-frequency non-linearities via a structurally regularized GBM, and (3) systematically integrating the baseline and residuals to mathematically reconstruct the absolute thermal conductivity. The complete pipeline is developed in Python, leveraging statsmodels for rigorous statistical inference and scikit-learn for advanced ensemble learning tasks.

### Data refinement and feature engineering

The empirical data utilized in this study is sourced from the comprehensive experimental compilation presented by^30^. This dataset consolidates experimental measurements from independent studies, establishing a robust ground truth for the thermal conductivity of metallic and metal-oxide nanofluids.

**Table 2:** Detailed statistical summary of the experimental datasets used in this study, classified by nanoparticle and base fluid type.

Nanoparticle	Base Fluid	N	Temp ($^\circ$C)	Vol. Frac. ($\phi$ %)	Size (nm)
		(Samples)	Min / Mean / Max	Min / Mean / Max	Min / Mean / Max
$\mathrm {Al_2O_3}$	Ethylene Glycol	48	20.0 / 35.0 / 50.0	0.50 / 1.63 / 3.00	11.0 / 68.7 / 150.0
$\mathrm {Al_2O_3}$	Transformer Oil	16	20.0 / 35.0 / 50.0	0.50 / 1.63 / 3.00	45.0 / 45.0 / 45.0
$\mathrm {Al_2O_3}$	Water	48	20.0 / 35.0 / 50.0	0.50 / 1.63 / 3.00	11.0 / 68.7 / 150.0
Aluminium	Ethylene Glycol	20	20.0 / 35.0 / 50.0	0.10 / 1.32 / 3.00	80.0 / 80.0 / 80.0
Aluminium	Transformer Oil	18	20.0 / 33.9 / 50.0	0.10 / 1.13 / 3.00	80.0 / 80.0 / 80.0
Aluminium	Water	20	20.0 / 35.0 / 50.0	0.10 / 1.32 / 3.00	80.0 / 80.0 / 80.0
Cu	Ethylene Glycol	20	20.0 / 35.0 / 50.0	0.10 / 1.32 / 3.00	80.0 / 80.0 / 80.0
Cu	Transformer Oil	20	20.0 / 35.0 / 50.0	0.10 / 1.32 / 3.00	80.0 / 80.0 / 80.0
Cu	Water	20	20.0 / 35.0 / 50.0	0.10 / 1.32 / 3.00	80.0 / 80.0 / 80.0
CuO	Ethylene Glycol	16	20.0 / 35.0 / 50.0	0.50 / 1.63 / 3.00	31.0 / 31.0 / 31.0
CuO	Transformer Oil	16	20.0 / 35.0 / 50.0	0.50 / 1.63 / 3.00	31.0 / 31.0 / 31.0
CuO	Water	16	20.0 / 35.0 / 50.0	0.50 / 1.63 / 3.00	31.0 / 31.0 / 31.0
Overall Statistics		**278**	**Total Materials:** 4 **Total Base Fluids:** 3		

The dataset encompasses a heterogeneous collection of nanofluid suspensions, defined by distinct particle materials dispersed across three base fluids (Water, Ethylene Glycol, and Transformer Oil). To ensure the predictive modeling framework captures the underlying thermodynamics rather than merely memorizing data points, the raw data was systematically transformed into physically meaningful features:

- **Target Variable** ($k_{ratio}$): Unlike traditional studies that predict absolute conductivity ($k_{nf}$), we transformed the target into the thermal enhancement ratio ($k_{ratio} = k_{nf} / k_{base}$). This normalization decouples the base fluid’s intrinsic thermodynamic properties from the nanoparticle effects, forcing the model to focus purely on the enhancement mechanisms.
- **Centered Temperature** ($T_c$): The operating temperature is mean-centered ($T_c = T - \bar{T}_{train}$) to reduce structural multicollinearity when generating polynomial and interaction terms within the GAM.
- **Inverse Particle Size** ($S^{-1}$): At the nanoscale, phenomena such as interfacial thermal resistance and available heat transfer area are heavily dependent on the specific surface area. Because specific surface area scales inversely with particle diameter, converting the raw size *S* into its inverse ($S^{-1}$) linearizes this critical geometric relationship, making it easier for the base GAM to capture the scaling physics.
- **Volume Fraction** ($\phi$): The volumetric concentration of nanoparticles within the base fluid (%), representing the primary driver of particle crowding and interaction effects.
- **Base Conductivity Proxy** ($\tilde{k}_{base}$): Standard models encode base fluids (e.g., Water, Oil) as categorical text labels, which mathematically prevents generalization to novel fluids. To overcome this, we replaced the categorical base fluid name with a continuous physical proxy by performing a Z-score normalization on the base fluid’s absolute thermal conductivity ($\tilde{k}_{base}$).
- **Categorical Material** ($\mathbb {I}_{Material}$): The specific nanoparticle material (e.g., Al$_2$O$_3$, CuO) remains categorically encoded, as it captures complex, discrete solid-state lattice effects that are difficult to parameterize continuously.

A stratified statistical summary of the dataset is presented in Table 2.

#### Variance stabilization (Box-Cox)

Experimental conductivity data typically exhibits heteroscedasticity, where the variance of the error terms increases alongside the magnitude of the physical target. To mitigate this and stabilize the variance, we apply a Box-Cox transformation to the target variable *Y* ($k_{ratio}$):

1 $$\begin{aligned} y^{(\lambda )} = {\left\{ \begin{array}{ll} \frac{Y^\lambda - 1}{\lambda } & \text {if } \lambda \ne 0 \\ \ln (Y) & \text {if } \lambda = 0 \end{array}\right. } \end{aligned}$$

The optimal transformation parameter, $\lambda$, is rigorously determined via Maximum Likelihood Estimation (MLE). Specifically, a profile log-likelihood function is evaluated across a standard continuous search domain (typically $\lambda \in [-2, 2]$). The specific $\lambda$ value that maximizes this likelihood, thereby yielding the most symmetric, normal-like distribution of the target variable, is selected. To maintain strict methodological integrity and prevent data leakage, the optimal $\lambda$ is computed exclusively using the training data partition, and this learned parameter is subsequently utilized to transform both the training and unseen validation sets.

#### RF-based outlier detection

Standard Z-score filtering relies on a stationary global mean, rendering it fundamentally inappropriate for heterogeneous, multi-fluid datasets where different base fluids exhibit drastically different thermodynamic baselines. To address this, we implemented a context-aware outlier detection mechanism driven by a RF regressor.

To ensure unbiased error estimation and prevent data leakage, the RF model was trained using a 5-fold cross-validation framework to generate robust out-of-fold predictions ($\hat{y}_{RF}$). The localized prediction error for each data point was then calculated as the residual $e_i = y_i - \hat{y}_{RF,i}$. Any observation exhibiting a residual magnitude exceeding 2.5 standard deviations ($|e_i| > 2.5\sigma$) from the residual mean was flagged as a severe experimental anomaly. These anomalous points were systematically removed from the dataset to prevent high-leverage measurement errors from contaminating the subsequent hybrid modeling architecture.

### The physics-guided hybrid architecture

The proposed framework predicts the transformed conductivity ratio $y^{(\lambda )}$ through a decoupled additive structure, combining a physics-based global estimator with a data-driven local corrector:

2 $$\begin{aligned} \hat{y}_{hybrid} = \underbrace{\hat{y}_{GAM}(\textbf{X}_{phys})}_{\text {Global Physics}} + \underbrace{\hat{y}_{GBM}(\textbf{X}_{all})}_{\text {Local Correction}}. \end{aligned}$$

The two components interact in a strictly asymmetric and sequential manner, designed so that each stage addresses a distinct class of physical behavior.

#### Stage 1: physics-informed generalized additive model (GAM)

To enable generalization to unseen fluid types, we depart from standard categorical encoding for the base fluid. Instead of learning discrete weights for specific fluid names (which fails for novel fluids), we utilize a continuous physical proxy: the normalized base conductivity.

The GAM formulation retains cubic B-splines for thermodynamic trends while integrating this physical proxy:

3 $$\begin{aligned} \begin{aligned} \hat{y}_{GAM} = \beta _0 + \underbrace{f_1(T_c) + f_2(\phi ) + f_3(S^{-1})}_{\text {Smooth Spline Terms}}&+ \underbrace{\beta _1(T_c \cdot \phi ) + \beta _2(T_c \cdot S^{-1})}_{\text {Linear Interactions}} \\&+ \underbrace{\beta _{phy} \cdot \tilde{k}_{base} + \sum _{j=1}^{J} \delta _j \mathbb {I}_{Material_j}}_{\text {Physical Proxy} \& \text {Categorical Material}}. \end{aligned} \end{aligned}$$

Where:

- $f_i(\cdot )$: Nonlinear smooth functions approximated by cubic B-splines, with optimal degrees of freedom $df^* \in \{2,\ldots ,6\}$ selected by minimizing the Akaike Information Criterion (AIC) and the Bayesian Information Criterion (BIC) during training.
- $\mu _{train}, \sigma _{train}$: The mean and standard deviation of the base fluid conductivity computed strictly from the training set to prevent data leakage.
- $\delta _j$: The learned fixed-effect coefficients corresponding to each nanoparticle material *j*, representing the discrete baseline shift in the predicted thermal conductivity ratio.

#### Stage 2: regularized residual learning via gradient boosting

The residuals from the GAM ($R = y^{(\lambda )} - \hat{y}_{GAM}$) contain both legitimate complex physical interactions and experimental noise. Given the limited dataset size, a standard Boosting approach risks memorizing high-frequency artifacts. To prevent this, we employ a Structurally Regularized GBM designed to isolate systematic bias while rejecting noise.

The residual estimator is defined as an ensemble of *M* heavily constrained weak learners:

4 $$\begin{aligned} \hat{R} = \sum _{m=1}^{M} \nu \cdot h_m(\textbf{X}; \theta _m)_{\Omega } \end{aligned}$$

Where $\Omega$ represents the strict regularization constraints imposed to ensure physical validity:

- **Depth Constraint** ($d=2$): Trees are restricted to simple pairwise interactions. This prevents the model from isolating specific data points, forcing it to learn broader physical patterns.
- **Stochastic Subsampling** ($\eta =0.8$): Each tree $h_m$ is trained on a random 80% subset of the data. This introduces randomness that prevents the ensemble from fixating on outliers.
- **Leaf Regularization** ($k_{Leaf}=6$): A terminal node is only valid if it contains at least 6 experimental samples (${\sim }2\%$ of the dataset), ensuring that any correction is statistically significant rather than a fluctuation.
- **Shrinkage** ($\nu =0.05$): A conservative learning rate dampens the update magnitude, ensuring stable convergence to a physical solution.

#### Stage 3: hybrid integration and physical reconstruction

To finalize the predictive pipeline, the outputs of the physics-informed GAM and the regularized GBM must be systematically combined and transformed back into the original physical domain. This interaction is structured as an additive synthesis in the transformed space, followed by an inverse mathematical mapping.

First, the global thermodynamic baseline predicted by the GAM ($\hat{y}_{GAM}$) and the localized non-linear correction generated by the GBM ($\hat{R}$) are summed to produce the final hybrid prediction in the Box-Cox transformed space:

5 $$\begin{aligned} \hat{y}_{hybrid} = \hat{y}_{GAM} + \hat{R} \end{aligned}$$

Next, the inverse Box-Cox transformation is applied to revert the stabilized prediction back to the thermal conductivity ratio scale:

6 $$\begin{aligned} \hat{y}_{ratio\_final} = \text {InvBoxCox}(\hat{y}_{hybrid}, \lambda ) \end{aligned}$$

Finally, the target physical property the thermal conductivity of the nanofluid ($\hat{k}_{nf}$) is mathematically reconstructed by multiplying the predicted ratio by the base fluid’s actual thermal conductivity ($k_{base}$):

7 $$\begin{aligned} \hat{k}_{nf} = \hat{y}_{ratio\_final} \times k_{base} \end{aligned}$$

This integration strategy enforces a strict division of labor between the statistical and machine learning components. The GAM acts as the primary governing equation, guaranteeing that the macroscopic physical laws (such as monotonic thermal enhancement with volume fraction) are structurally respected across the entire prediction space. Conversely, the GBM acts exclusively as a localized perturbation function. Because it is trained solely on the GAM’s residuals, the GBM only activates in complex regimes where purely additive splines fail, such as the Kapitza resistance degradation observed at ultra-small nanoparticle diameters. By combining them additively before the inverse transformation, the framework mathematically guarantees that the machine learning algorithm can locally refine the prediction without overwriting the fundamental thermodynamic baseline. The sequential execution of this entire three-stage architecture is summarized as a computational pseudocode in Algorithm 1.

**Algorithm 1:**
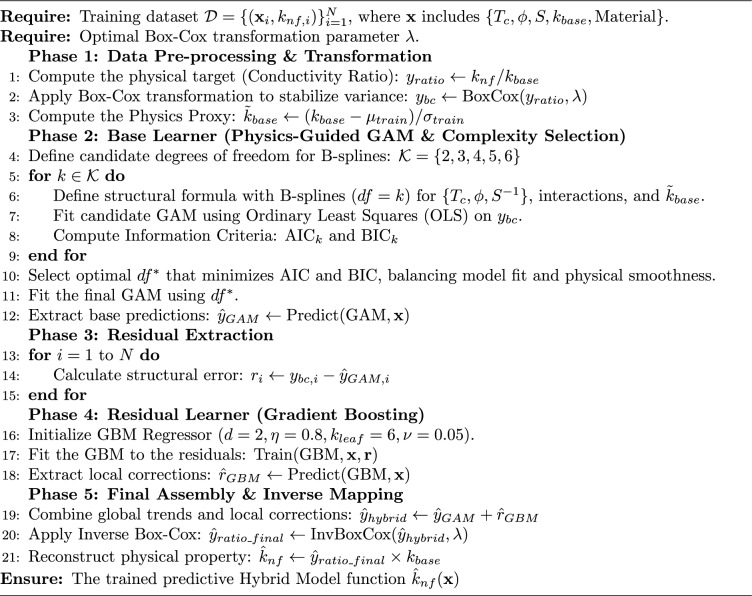
Training Protocol for the Physics-Informed Hybrid Model (GAM+GBM)

### Benchmarking protocol

To validate the hybrid model, we compared it against standard ML regressors. Hyperparameters for each model were optimized using Grid Search with 5-fold Cross-Validation (CV). The competing models include:

- *Ensemble Methods* RF, GBM, Bagging.
- *Kernel Methods* GP utilizing a grid search strategy to identify the optimal covariance function among multiple candidates (RBF, Matern, Rational Quadratic), SVR.
- *Deep Learning* Multi-layer Perceptron (MLP) (ReLU activation).
- *Tree-Based* Standard DT.

### Generalization protocol: leave-one-group-out (LOGO)

To rigorously assess the framework’s ability to generalize to unseen thermophysical environments, we implemented a LOGO validation protocol. Standard *k*-fold cross-validation often yields over-optimistic performance estimates due to information leakage between similar samples. In contrast, the LOGO protocol systematically excludes an entire class of Base Fluids from the training set to simulate a “blind” prediction scenario.

**Fig. 2:**
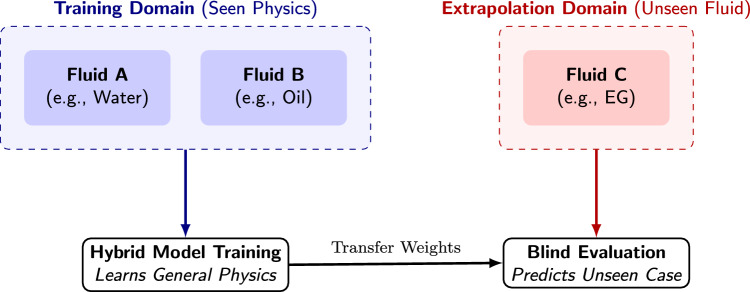
Conceptual illustration of the LOGO protocol. The model is trained on a subset of fluids (Training Domain) and evaluated on a completely excluded fluid (Extrapolation Domain) to test strictly for physical generalization rather than interpolation.

As illustrated in Fig. 2, the algorithm iterates through the unique base fluids (Water, Ethylene Glycol, Transformer Oil). To ensure algorithmic transparency, the exact statistical composition of these groupings must be noted. The dataset is not uniformly distributed among the base fluids; Water constitutes the majority of the experimental samples (37.4%), followed by Ethylene Glycol (37.4%) and Transformer Oil (25.2%). Consequently, the LOGO iterations represent varying degrees of extrapolation severity. Furthermore, care was taken to verify that the distinct categorical nanoparticle materials (e.g., Al$_2$O$_3$, CuO) are sufficiently distributed across multiple base fluids. This ensures that when a specific base fluid is withheld, the model is not inadvertently blinded to an entire class of nanoparticle solid-state physics, isolating the extrapolation test purely to the fluid’s thermodynamic domain. In each iteration, $N-1$ fluids constitute the “Training Domain”, while the $N^{th}$ fluid constitutes the “Unseen Extrapolation Domain”. Crucially, the Hybrid GAM+GBM model utilizes normalized physical proxies (e.g., base conductivity deviation) rather than explicit categorical labels during this phase, forcing the model to learn universal heat transfer mechanisms rather than memorizing fluid identities.

This test determines whether the algorithm has successfully decoupled the base fluid properties from the nanoparticle enhancement ratio, a critical requirement for deploying ML models in novel engineering applications.

## Results and discussion

### Data transformation and quality control analysis

Before training the predictive models, the data preprocessing pipeline was rigorously evaluated. The Box-Cox transformation parameter, $\lambda$, was initially optimized via maximum likelihood estimation without boundaries. Through five-fold cross-validation, the unconstrained optimal value was determined to be $-5.2840 \pm 0.3107$. Although this highlights a complex inverse relationship, the extremity and variance of this parameter demonstrate numerical sensitivity and result in an unphysical scaling of the target variable. Consequently, to maintain physical interpretability and robust model generalization, the algorithm was strictly constrained to an optimization interval of $\lambda \in [-2, 2]$.

Subsequently, the Random Forest-based outlier detection scan was executed on the transformed feature space. Using the baseline threshold of $\pm 2.5\sigma$, the algorithm identified 9 data points with excessive residual Z-scores. These points were flagged as non-physical experimental artifacts and removed, resulting in a final high-quality dataset of 269 samples for the cross-validation stage. To verify the robustness of this filtering criterion, a sensitivity analysis was conducted using alternative thresholds of $\pm 2.0\sigma$ and $\pm 3.0\sigma$. As detailed in Table 3, the predictive accuracy of the Hybrid model remained highly stable across all configurations (with Root Mean Square Error (RMSE) fluctuating by less than 0.0002 W/mK). This confirms that the model’s strong performance is not an artifact of a specific outlier threshold, ensuring high reproducibility.

**Table 3:** Sensitivity analysis of the Hybrid model’s performance across different outlier detection thresholds.

Threshold	Outliers removed	Remaining samples	Hybrid RMSE (W/mK)
$\pm 2.0\sigma$	12	266	0.00476
$\pm 2.5\sigma$ (Baseline)	9	269	0.00482
$\pm 3.0\sigma$	4	274	0.00488

### Predictive performance benchmark

To ensure the base GAM possessed sufficient mathematical flexibility to capture complex nonlinear dependencies, such as the multistage percolation behavior of the nanoparticles, a degrees of freedom (*df*) selection analysis was conducted. The model structure was evaluated across $df \in [2, 7]$ using the AIC and BIC. The cubic configuration ($df=3$) yielded (AIC = -1603.26 and BIC = -1549.1). However, increasing the flexibility to $df=4$ resulted in a statistically significant improvement, minimizing the information criteria (AIC= -1607.92, BIC= -1550.41). For configurations of $df \ge 5$, the information criteria flatlined, indicating that additional knots provided no further explanatory power. Consequently, $df=4$ was selected as the optimal, parsimonious configuration for the spline based physical predictors.

**Fig. 3:**
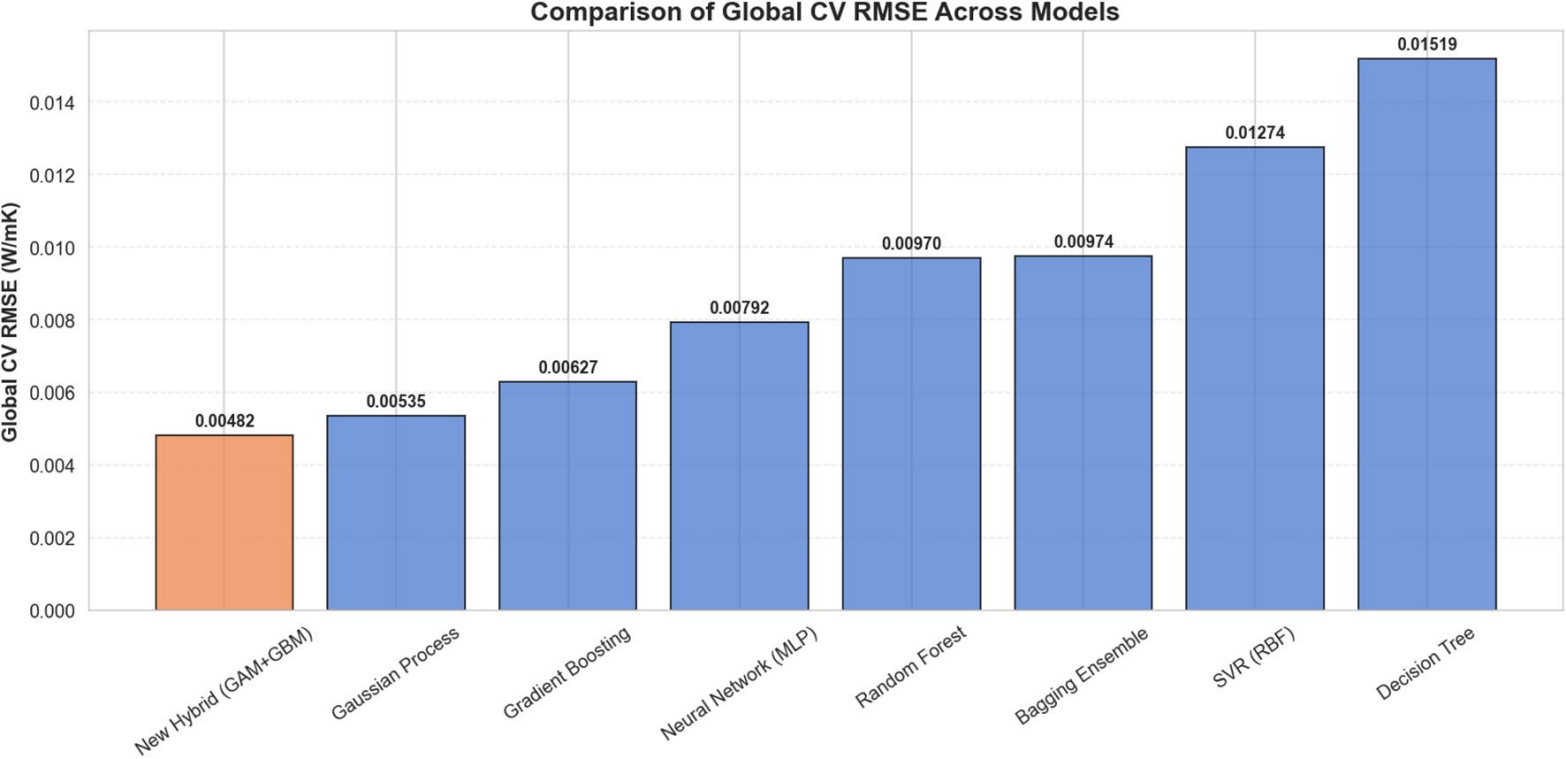
Comparative analysis of RMSE across all tested models. The Proposed Hybrid Model (0.0048) achieves the best performance, surpassing the opaque GBM optimum (0.0063) by effectively decoupling global physical trends from local data irregularities.

The predictive capabilities of the proposed Physics-Guided Hybrid framework were evaluated against seven established machine learning architectures. The optimal hyperparameters for each model, determined via grid search, are detailed in Table 4. The comparative performance, measured by RMSE, Mean Absolute Error (MAE), and Coefficient of Determination ($R^2$), is listed in Table 5 and visualized in Fig. 3.

**Table 4:** Optimal hyperparameters selected for each model via 5-Fold Cross-Validation Grid Search.

Model Architecture	Configuration & Hyperparameters
Hybrid (GAM+GBM)	**Base:** GAM (Splines $df=4$ on Temp, Vol, Inv_Size + Interactions) **Residuals:** GBM ($M=100,\, k_{leaf} = 6,\, d=2,\, \nu =0.05$)
GBM	$M=300$, $k_{leaf} = 1$ ,$\nu =0.1$, $d=3$, Loss=Squared Error
GP	Kernel: Rational Quadratic ($\alpha =1.0, l=1.0$) + White Noise
RF	$M=500$, $d=10$, $k_{min}=2$, $N_{feat}=1.0$ (All Features)
Bagging Ensemble	Base=DecisionTree, $M=100$, $max\_samples=1.0$
SVR (RBF)	$C=10$, $\epsilon =0.01$, $\gamma =scale$
Neural Network (MLP)	Solver=LBFGS, $N_{iter}=5000$, Layers=(16, 8), $\alpha =0.01$, Act=ReLU
DT	$d=10$, $k_{min}=2$, Criterion=Squared Error

**Table 5:** Comparative predictive performance sorted by precision.

Rank	Model	RMSE	MAE	$\boldsymbol{R^2}$
1	Hybrid (GAM+GBM)	0.00482	0.00317	0.9996
2	GP	0.00535	0.00384	0.9995
3	GBM	0.00627	0.00425	0.9993
4	Neural Network (MLP)	0.00793	0.00588	0.9988
5	RF	0.00970	0.00650	0.9983
6	Bagging Ensemble	0.00645	0.00645	0.9983
7	SVR (RBF)	0.01274	0.00924	0.9973
8	DT	0.01519	0.01025	0.9959

The Physics-Guided Hybrid model achieves the lowest error, outperforming standard black-box ensembles.

The benchmarking results (summarized in Table 5) reveal a distinct hierarchy in model performance:

1. *Tier 1 (Physics-Guided Precision)* The Hybrid GAM+GBM model achieved the lowest global error ($RMSE \approx 0.0048$ W/mK, $MAE \approx 0.0032$ W/mK, $R^2 \approx 0.9996$), decisively outperforming both the GP ($RMSE \approx 0.0054$ W/mK) and the standard GBM model ($RMSE \approx 0.0063$ W/mK). This result is significant: it demonstrates that explicitly modeling the physical base trends via splines provides a superior starting point for the boosting algorithm compared to learning entirely from scratch.
2. *Tier 2 (Probabilistic & Strong Ensembles)* GP regression led the second tier ($RMSE \approx 0.0054$ W/mK, $MAE \approx 0.0038$ W/mK), validating its capacity to handle the complex, non-stationary nature of nanofluid thermal properties. It was closely followed by standard, unguided ensemble methods, namely the standalone GBM ($RMSE \approx 0.0063$ W/mK) and the Bagging Ensemble ($RMSE \approx 0.0065$ W/mK).
3. *Tier 3 (Lower Capacity & High Variance Baselines)* This tier encompasses the Neural Network (MLP), RF, and SVR, which exhibited moderate predictive errors ($RMSE \approx 0.0079 - 0.0127$ W/mK). The standalone DT exhibited the highest overall error ($RMSE \approx 0.0152$ W/mK, $MAE \approx 0.0103$ W/mK), confirming that a single, unregularized tree lacks the inductive bias necessary to model smooth thermodynamic curves without the structural stabilization provided by the Hybrid framework.

**Fig. 4:**
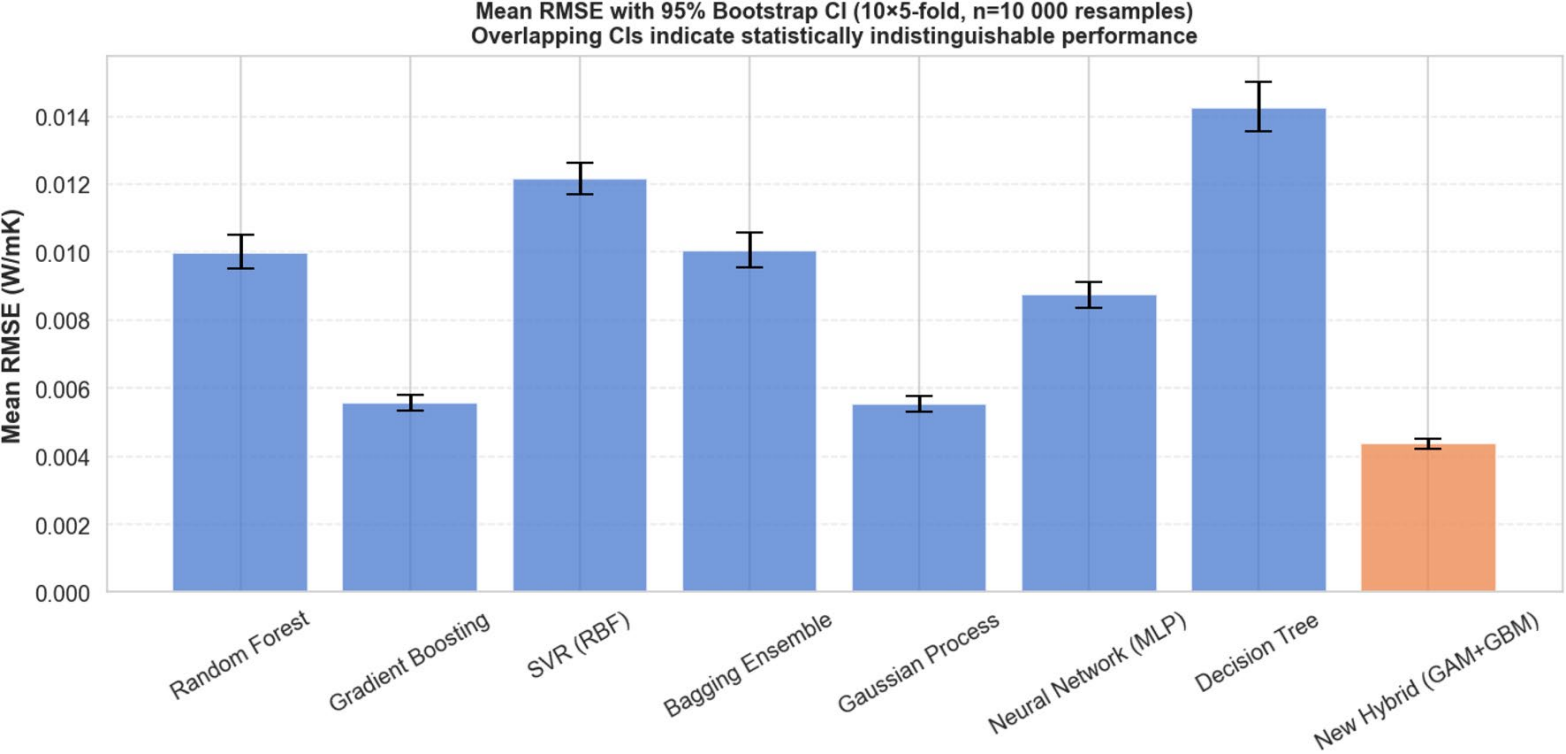
Mean RMSE with 95% Bootstrap Confidence Intervals (10,000 resamples) derived from a repeated 10$\times$5-fold cross-validation. The strict separation between the Hybrid model’s CI (orange) and those of the baseline architectures visually corroborates the statistical significance established by the Nadeau-Bengio corrected tests.

While point estimates of predictive error (such as a single cross-validated RMSE) provide a general hierarchy of model performance, they do not account for the variance inherent in finite datasets. To rigorously evaluate the superiority of the proposed Hybrid (GAM+GBM) model, a repeated cross-validation strategy ($10\times 5$-fold) was executed. As presented in Table 6, the Hybrid model achieved the lowest Mean RMSE of 0.004382 W/mK. To determine if this advantage was statistically significant, a Nadeau-Bengio corrected paired t-test was performed against each baseline model. The Nadeau-Bengio correction was specifically utilized to counteract the inflated Type-I error rates typical of standard t-tests evaluated on overlapping training folds. The results confirm that the Hybrid model statistically outperforms all benchmarked architectures ($p < 0.01$). Even against the highest performing purely data-driven baselines the Gaussian Process ($p = 0.0088$) and Gradient Boosting ($p = 0.0050$) the Hybrid model demonstrates a statistically significant reduction in error. Furthermore, the effect size of this reduction is substantial, yielding a Cohen’s *d* magnitude greater than 1.6 against the closest competitors, and exceeding 4.0 against standard ensemble methods like Random Forest. To visually corroborate these findings, a 10,000-iteration bootstrap resampling was performed on the fold-level RMSE values to compute the 95% Confidence Intervals (CIs). As illustrated in the resulting forest plot (Fig. 4), the 95% CI of the Hybrid model [0.004233, 0.004532] does not overlap with the CI of any other model. This clear separation confirms that the performance advantage of explicitly decoupling the global physical trends from the localized residuals is robust, significant, and strictly verified against statistical noise.

**Table 6:** Repeated cross-validation (10$\times$5-fold) and statistical significance testing.

Model	Mean RMSE (W/mK)	Bootstrap 95% CI	Mean $R^2$	*p*-value (NB-corr.)	Cohen’s *d*
DT	0.014266	[0.013567, 0.015009]	0.9964	$<0.0001$	$-5.2736$
SVR (RBF)	0.012153	[0.011697, 0.012640]	0.9975	$<0.0001$	$-6.0411$
Bagging Ensemble	0.010050	[0.009548, 0.010583]	0.9982	$<0.0001$	$-4.1363$
RF	0.009988	[0.009507, 0.010500]	0.9983	$<0.0001$	$-4.2383$
Neural Network (MLP)	0.008740	[0.008366, 0.009128]	0.9986	$<0.0001$	$-4.1207$
GBM	0.005563	[0.005336, 0.005797]	0.9994	0.0050	$-1.6574$
GP	0.005547	[0.005311, 0.005782]	0.9994	0.0088	$-1.6127$
Hybrid (GAM+GBM)	**0.004382**	**[0.004233, 0.004532]**	**0.9996**	**–**	**–**

The proposed Hybrid model is compared against each baseline using a Nadeau-Bengio corrected paired t-test ($H_0$: $\text {RMSE}_{\text {Hybrid}} = \text {RMSE}_{\text {Baseline}}$). The bootstrap confidence intervals (10,000 resamples) illustrate the tight variance of the proposed architecture.

### Visual validation: parity analysis and model fidelity

**Fig. 5:**
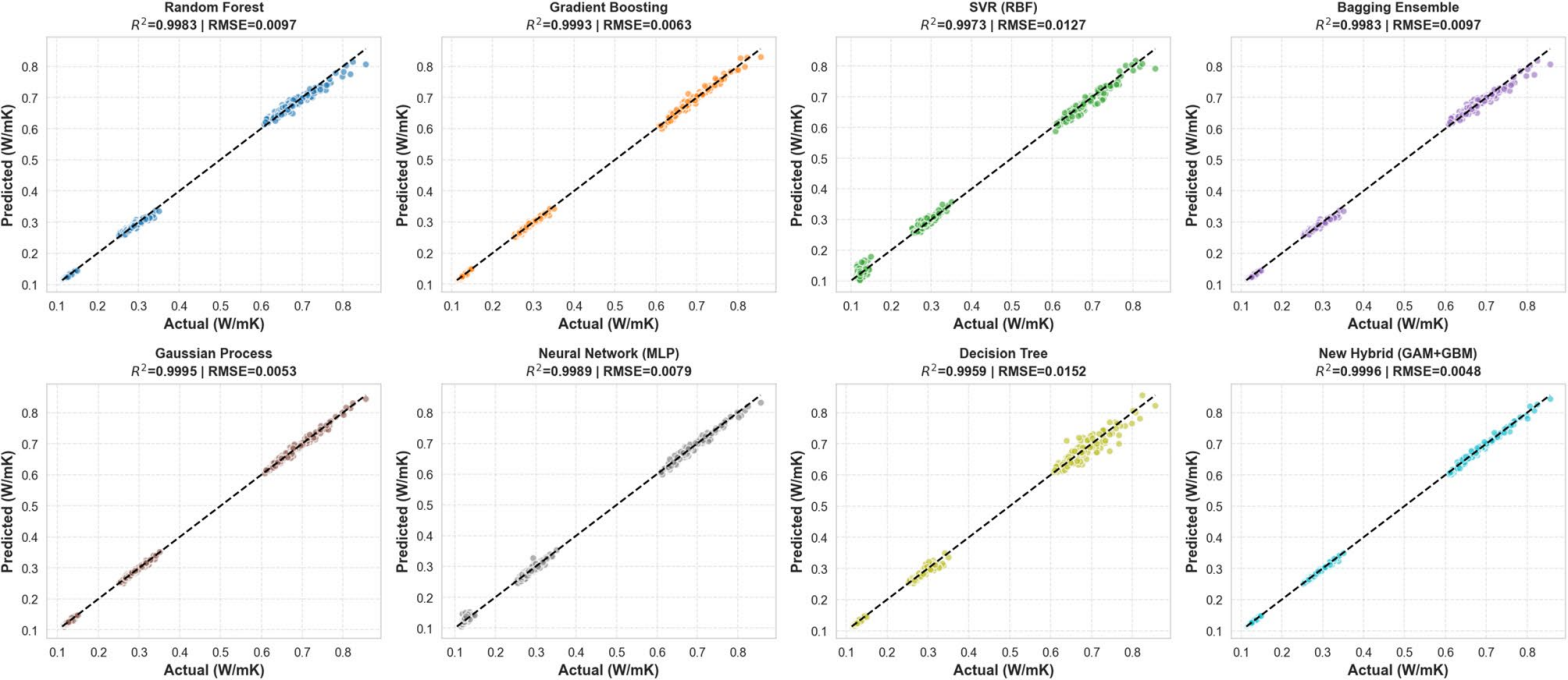
Parity plots comparing predicted versus experimental thermal conductivity for all eight benchmarked architectures. The dashed line indicates perfect agreement. The Hybrid (GAM+GBM) model demonstrates high-fidelity adherence to the diagonal, effectively mitigating the high-conductivity variance observed in SVR and MLP while eliminating the discretization errors of the single DT.

To substantiate the statistical metrics, we performed a visual diagnostic of the model alignment via parity plots, as presented in Fig. 5. These plots map the predicted thermal conductivity ($\hat{y}$) against the experimental ground truth (*y*), where the dashed $45^\circ$ diagonal represents the line of perfect concordance ($\hat{y} = y$).

A comparative inspection of the regression topologies reveals three critical insights:

- *Superiority of Physics-Guided Boosting (Hybrid vs. GBM)* The *Hybrid (GAM+GBM)* model (bottom right) exhibits an exceptionally tight coalescence around the diagonal, demonstrating visibly lower dispersion than the pure GBM model (top, second from left). While the pure GBM achieves a respectable $R^2 \approx 0.9993$, the Hybrid framework surpasses it with an $R^2 \approx 0.9996$ and the lowest global error (RMSE = 0.0048 W/mK). This confirms that explicitly modeling the physical baseline via the GAM prevents the boosting stage from struggling with global trends, allowing the Hybrid framework to outperform standard black-box ensembles.
- *Failure of Discrete Partitioning (DT)* The standalone DT (bottom row, third column) displays significant variance and characteristic “discretization artifacts” (vertical clustering). This visual scatter ($RMSE=0.0152$ W/mK) highlights the fundamental inability of a single tree to approximate smooth, continuous thermodynamic gradients without the stabilizing influence of the GAM backbone.
- *Heteroscedasticity in Standard ML* The *SVR* and *Neural Network (MLP)* models exhibit noticeable heteroscedasticity the prediction spread widens as thermal conductivity increases ($>0.7$ W/mK). In contrast, the Hybrid model maintains uniform homoscedasticity across the entire domain, suggesting it is more robust to the high-variance regimes typical of water-based nanofluids at elevated temperatures.

### Computational complexity and efficiency analysis

In addition to predictive precision, the practical deployment of machine learning architectures in materials science depends on their computational efficiency. To evaluate this, the average training and inference time per cross validation (10$\times$5 fold) was recorded for all models. A paired t-test was then conducted to determine if the computational overhead of the proposed Hybrid framework significantly differed from the baseline models. The results are summarized in Table 7.

**Table 7:** Computational training time per cross-validation (10$\times$5 fold).

Model	Mean Time (s)	Std Time (s)	p-value	Hybrid is...
DT	0.0062	0.0034	$<0.0001$	Slower
SVR (RBF)	0.0063	0.0030	$<0.0001$	Slower
GBM	0.3276	0.1088	$<0.0001$	Slower
Bagging Ensemble	0.3790	0.0971	$<0.0001$	Slower
Neural Network (MLP)	0.4457	0.0498	$<0.0001$	Slower
RF	1.3276	0.2364	$<0.0001$	Faster
GP	2.9359	1.6172	$<0.0001$	Faster
New Hybrid (GAM+GBM)	**0.7016**	**0.0574**	**–**	**–**

Statistical significance was determined using a paired t-test comparing the proposed Hybrid model against each baseline ($H_0$: $\text {Time}_{\text {Hybrid}} = \text {Time}_{\text {Baseline}}$).

The analysis reveals a clear Pareto frontier governing the trade-off between computational speed and predictive accuracy. The strictly algorithmic approaches, such as the DT and SVR models, were exceptionally fast, executing in approximately 0.006 seconds. However, this speed comes at the severe cost of high predictive error (RMSE $> 0.0120$ W/mK) and theoretical opacity. The proposed Hybrid (GAM+GBM) model required an average computational time of $0.7016 \pm 0.0574$ seconds per fold. While statistically slower than the standalone GBM (0.3276 seconds, $p < 0.0001$), this $\sim 0.37$ second overhead is mathematically expected. The Hybrid architecture requires a sequential two-stage optimization: first estimating the spline coefficients via highly efficient OLS for the GAM, and subsequently growing the regression trees for the GBM residual corrector. Given the sub-second execution time on standard CPU hardware, this marginal increase in computational cost is negligible.

Crucially, the Hybrid framework proved to be significantly faster and computationally lighter than the other high-capacity baseline models. It executed in nearly half the time of the Random Forest ensemble (1.3276 seconds, $p < 0.0001$) and was more than four times faster than GP Regression (2.9359 seconds, $p < 0.0001$). This speed advantage over GP is a critical determinant for future scalability. While GP models scale cubically ($\mathcal {O}(N^3)$) with dataset size, rendering them mathematically prohibitive for massive industrial datasets, the underlying mathematical operations of the Hybrid model scale highly efficiently. The computational complexity of the Hybrid model is strictly bounded by the additive spline evaluations and the regularized, shallow depth ($d=2$) of the boosting ensemble ($\mathcal {O}(M \cdot N \log N)$, where *M* is the number of estimators).

Furthermore, while training times are relevant for model development, real-world deployment scalability is ultimately dictated by inference latency. Evaluating novel nanofluid configurations through the trained Hybrid architecture merely requires passing inputs through continuous basis functions and traversing a small ensemble of shallow decision trees. This yields near-instantaneous execution times ($\sim$milliseconds per prediction) without the necessity for GPU acceleration, making the proposed PGML framework highly scalable and uniquely suited for integration into real-time thermal management control loops and large-scale digital twin simulations.

### Transcending the accuracy-interpretability frontier

A distinct and critical finding of this study is the inversion of the classical “accuracy vs. interpretability” trade-off. Conventional wisdom suggests that gaining transparency requires a sacrifice in predictive power. However, our benchmarking reveals that the Hybrid (GAM+GBM) model not only matches but marginally outperforms the opaque GBM optimum (*RMSE* : 0.0048 vs 0.0063 W/mK).

This result suggests that for thermophysical data, “Black-Box” models suffer from a lack of inductive bias, they must waste capacity learning basic physical laws (e.g., that conductivity increases with temperature) from scratch. By offloading these known truths to the GAM component, the GBM residuals can focus exclusively on complex, high-frequency anomalies. Consequently, the Hybrid framework offers a Pareto improvement: it provides the highest available precision while retaining the granular explainability of a statistical model.

### Deciphering the residuals: analysis of the machine learning correction

A key advantage of the Hybrid (GAM+GBM) framework is its ability to explicitly isolate and analyze the residual correction signal. By construction, the GBM operates only on the residuals of the baseline GAM, allowing its predictions to be interpreted as structured deviations from the parametric trends. To identify the physical regimes in which these deviations arise, SHAP (Shapley Additive Explanations) is employed to quantify the contribution of each input variable to the learned correction.

#### SHAP feature importance of the residuals

**Fig. 6:**
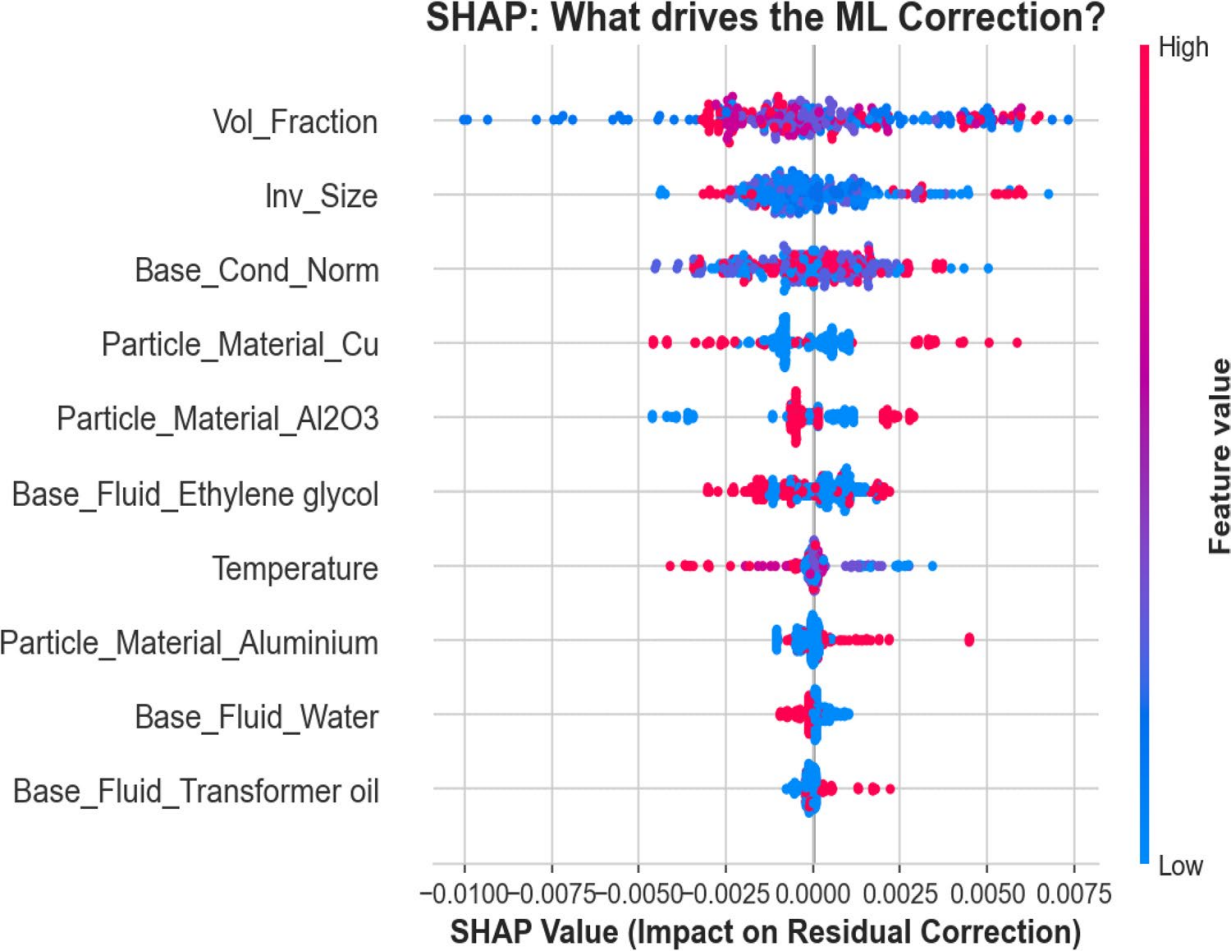
SHAP summary plot for the residual learning component. Volume fraction is the dominant driver of the ML (GBM) correction. Positive SHAP values correspond to an upward adjustment of the base GAM prediction, while negative values indicate a downward correction.

Figure 6 presents the SHAP summary plot for the residual learner. Unlike conventional analyses that rank primary physical drivers, this visualization ranks the drivers of the model correction, revealing where the GAM approximation required non-parametric refinement.

Volume fraction (Row 1) and inverse particle size (Inv_Size, Row 2) dominate the corrections, indicating that the GAM’s additive splines cannot fully capture the complex, nonlinear behaviors emerging from particle crowding and extreme geometric limits. A secondary tier, comprising the base conductivity proxy (Base_Cond_Norm, Row 3) and specific material or fluid identifiers (Rows 4–6), introduces moderate, localized adjustments.

Crucially, temperature (Row 7) and the remaining categorical baselines (Rows 8–10) exhibit near-zero SHAP impact. This mathematically validates the hybrid architecture: the base GAM successfully isolated and captured the primary thermodynamic temperature dependency and fluid baselines, leaving the boosting algorithm to focus exclusively on complex geometric and concentration-driven nonlinearities.

- *Correction Mechanism (Volume Fraction)* The SHAP distribution for volume fraction exhibits a clear, diverging directional trend based on concentration levels. High concentrations (red points) are predominantly associated with positive SHAP values (e.g., 0.0025 to 0.0075). This indicates that the GBM systematically increases the predicted conductivity in high-loading regimes, compensating for the baseline GAM’s tendency to under-predict when particle interactions are strong. Conversely, low concentrations (blue points) are predominantly clustered in the negative SHAP region (between $-0.005$ and 0.000). This indicates that the GBM applies a systematic downward correction in low-loading regimes, implying that the baseline GAM slightly over-predicts thermal conductivity when nanoparticle presence is minimal.
- *Geometric Correction (Inverse Particle Size)* Unlike volume fraction, the Inv_Size feature does not exhibit a strong or systematic correction trend. The topological dispersion of corrections is heterogeneous and largely symmetric around the zero line, lacking a definitive directional bias. This indicates that the GAM’s global size spline adequately captured the primary geometric dependencies. Consequently, the GBM does not identify any fundamental physical misalignment to correct, applying only minor, random adjustments rather than a structured geometric recalibration.
- *Thermodynamic Calibration (Temperature)* Although Temperature (Temp_C) exerts a very small overall magnitude of correction compared to other features, its SHAP distribution reveals a specific directional gradient. Very high temperatures (red points) consistently correspond to negative SHAP values. This indicates that the base GAM slightly over-predicts thermal conductivity at elevated temperatures, leading the GBM residual learner to apply a minor downward (negative) correction in these high-temperature regimes.

#### Topology of the correction signal

**Fig. 7:**
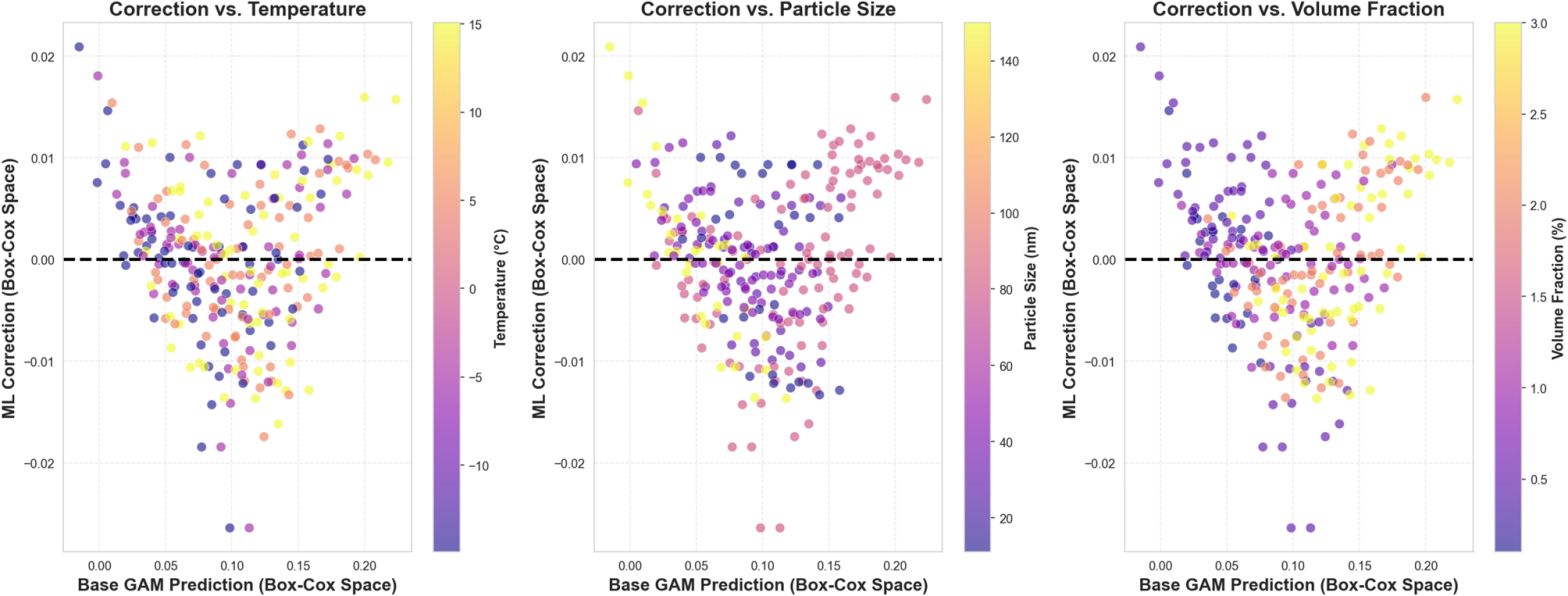
Topological analysis of the machine learning correction plotted against the base GAM prediction. The correction signal is unstructured and tightly bound with respect to temperature (left), indicating sufficient GAM capture. Conversely, a clear, systematic positive divergence is observed for high volume fractions (right), mathematically demonstrating the GBM’s role in resolving dense-regime nonlinearities that standard additive models under-predict.

To visualize how the residual learner’s interventions are distributed across the prediction space, Fig. 7 plots the magnitude of the ML (GBM) correction against the base GAM prediction, with the scatter points colored by the magnitude of key physical variables. This topological analysis corroborates the SHAP findings and reveals exactly where the foundational splines required non-parametric refinement:

- *Temperature (Left Panel — Thermodynamic Sufficiency)* The temperature panel exhibits a tight, symmetric dispersion clustered closely around the zero-correction line. While the SHAP analysis identified a subtle calibration gradient, the lack of any massive, color-stratified structural deviation in this macroscopic view confirms that the correction magnitude is negligibly impacted by temperature extremes. This visually demonstrates that the GAM’s additive splines successfully captured the bulk thermodynamic enhancement, leaving the GBM to make only minor, localized adjustments.
- *Particle Size (Middle Panel — Localized Refinement)* The particle size panel shows a heterogeneous dispersion of corrections without a single sweeping topological detachment. The mixed color distribution vertically surrounding the zero line perfectly reflects the SHAP geometric insights: the GAM captures the global inverse-size trajectory, while the GBM applies localized, bidirectional vertical refinements, such as the downward (negative) corrections for ultra-small particles.
- *Volume Fraction (Right Panel — Systematic Divergence)* A distinct, systematic topological deviation emerges exclusively in the right panel. In exact agreement with the SHAP mechanism, as the base GAM prediction increases, samples characterized by high volume fractions (lighter points) consistently detach from the zero line and arc strongly into the positive correction territory. Conversely, lower concentration samples (darker points) remain tightly bound near or slightly below the zero line. This provides stark visual proof of the hybrid model’s core value: the GBM actively and systematically compensates for the purely additive GAM’s tendency to underestimate thermal conductivity in dense, highly interacting nanoparticle regimes.

### Interpretation of physical dependencies and data sparsity

The true value of the Hybrid architecture lies in its ability to decouple global thermodynamic trends from local, data-driven corrections. Unlike opaque ensemble methods, we can visualize the learned partial dependence profiles to verify physical consistency.

**Fig. 8:**
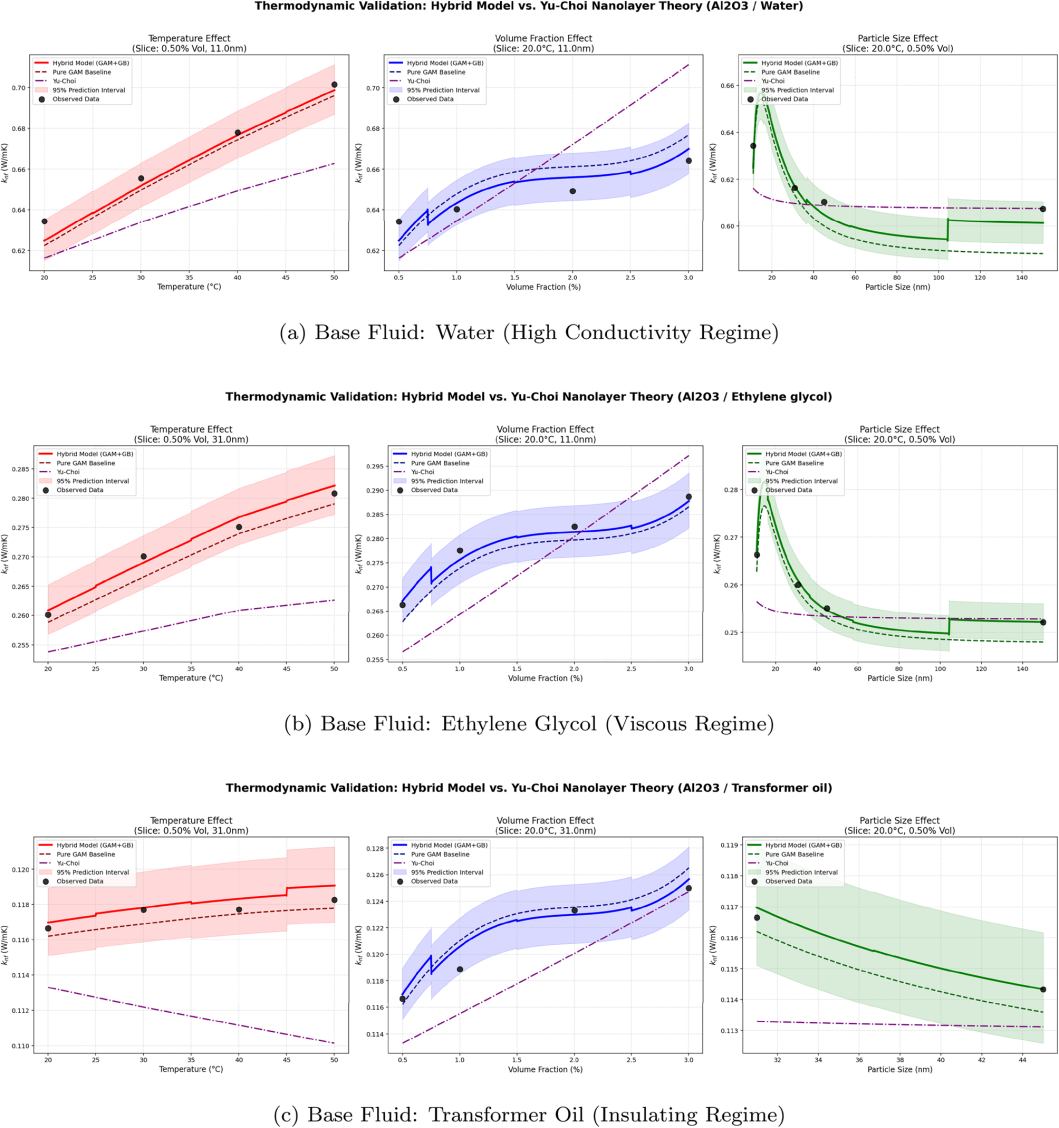
Thermodynamic validation plots generated by the Hybrid framework for Al$_2$O$_3$ nanoparticles across three distinct base fluids. The Hybrid (GAM+GBM) model is benchmarked against its own purely continuous GAM baseline and the theoretical Yu-Choi (2003) interfacial nanolayer model to demonstrate physical consistency and isolate algorithmic correction artefacts.

To isolate the effects of the thermodynamic and geometric variables, marginal factor analysis profiles (Fig. 8) were generated. These plots isolate the specific effect of Temperature, Volume Fraction, and Particle Size by anchoring the theoretical model curve to a representative real-world experimental setup for each specific base fluid (using Al$_2$O$_3$ nanoparticles). To rigorously validate the physical consistency of the framework, the Hybrid Model predictions are plotted alongside both the underlying Pure GAM Baseline and the modern Yu and Choi^31^ Interfacial Nanolayer Theory. To ensure statistical transparency, 95% prediction intervals (shaded regions) and all corresponding raw experimental data points are overlaid. These profiles reveal three distinct physical behaviors:

1. *Temperature Effect (Thermodynamic Enhancement)* All three base fluids exhibit a monotonic, near-linear increase in thermal conductivity with temperature, with observed points aligning closely with the predicted trend. The absolute magnitude is governed by the base fluid: Water rises from $\approx$0.623 to 0.700 W/mK, Ethylene Glycol from $\approx$0.260 to 0.283 W/mK, and Transformer Oil shows an almost negligible gradient ($\approx$0.1168 to 0.1185 W/mK) with wider prediction intervals. Physically, this positive gradient demonstrates strict thermodynamic consistency, perfectly aligning with the intensification of Brownian motion; as temperature rises, the increased kinetic energy and micro-convection of the nanoparticles facilitate faster thermal transport. Notably, the Hybrid model successfully captures this underlying physical dynamic, closely tracking the theoretical temperature dependence established by the Yu-Choi model in at minimum Water and Ethylene glycol base fluids.
2. *Volume Fraction Effect (Concentration Dynamics)* A consistent non-monotonic discontinuity near $\phi \approx 0.75$–1.0% is visible across all fluids. By plotting the underlying models separately, it is explicitly evident that this is an artefact of the GBM correction; the purely physics-guided GAM baseline maintains a smooth, continuous trajectory, while the GBM induces a discrete jump to bridge the data sparsity between distinct experimental clusters. Beyond this region, thermal conductivity increases monotonically with concentration. The Hybrid and GAM models both predict a weaker enhancement than the Yu-Choi nanolayer theory, particularly at higher loadings, suggesting that the nanolayer mechanism alone overestimates the experimental enhancement in these datasets. Prediction intervals narrow appropriately at higher loadings where data coverage is denser.
3. *Particle Size Effect (Interfacial Resistance and Sparsity)* For Water and Ethylene Glycol, the observed data indicates high thermal conductivity at the smallest measured particle size ($S \approx 11$ nm), the Hybrid and Pure GAM models predict a sharp, unobserved peak (at 15 nm) in conductivity occurring within the data gap between 11 nm and 30 nm. The inclusion of the theoretical Yu-Choi curve validates the general physical trend of enhanced macroscopic conductivity at smaller particle sizes driven by a drastically higher surface-area-to-volume ratio and a larger relative volume fraction of the highly conductive interfacial nanolayer. However, the Yu-Choi model predicts a smooth, monotonic decay and does not validate the drastic spike predicted by the machine learning models. Past this peak, the Pure GAM baseline successfully captures the continuous decay as particles enlarge toward 45 nm. Two important caveats must be applied to these model predictions. First, the Hybrid model exhibits a sharp step discontinuity near $S \approx 105$ nm, which is clearly identifiable as a GBM correction artifact when contrasted with the smooth Pure GAM baseline. Second, the complete absence of experimental measurements between 11 nm and 30 nm coincides precisely with the predicted conductivity spike, causing the 95% prediction intervals to widen substantially in this region. Consequently, the dramatic curve shape in this gap must be interpreted with caution as a highly uncertain model interpolation, rather than a theoretically validated or data-dense optimum.

#### Synergistic interaction analysis: temperature and concentration

**Fig. 9:**
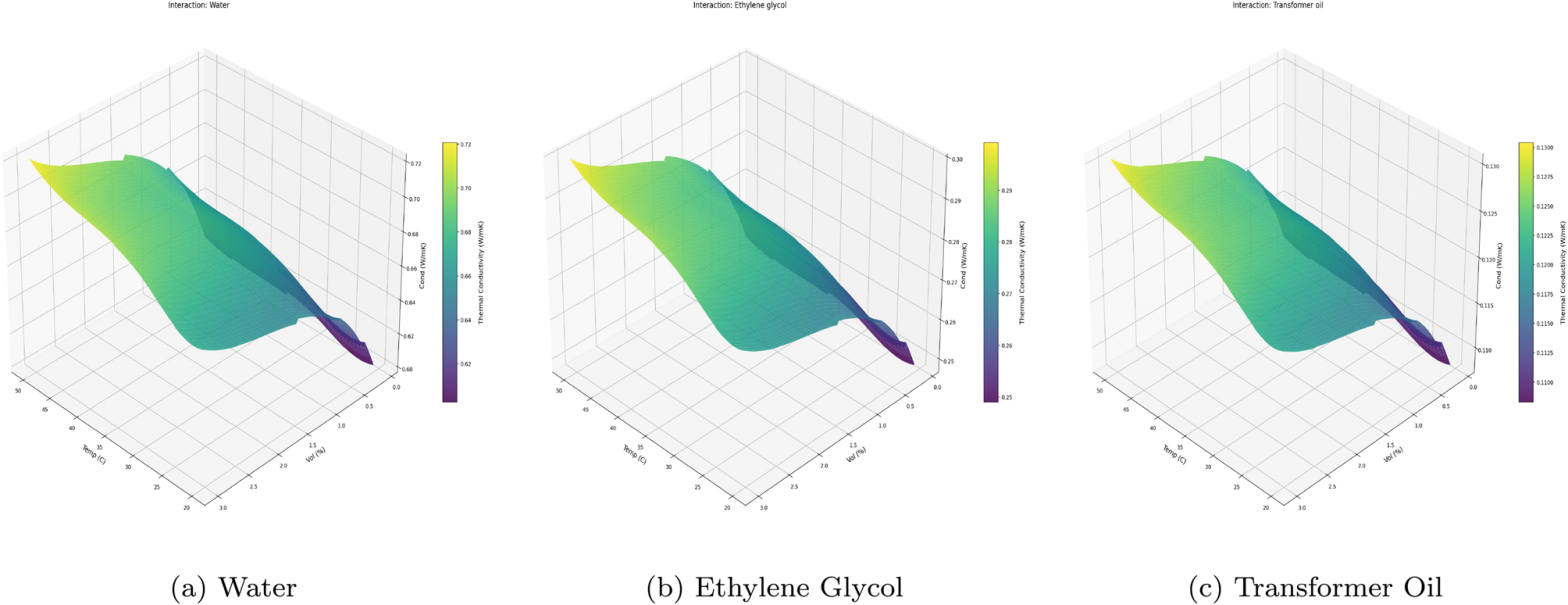
3D Response Manifolds illustrating the synergistic coupling between Temperature and Volume Fraction for Al$_2$O$_3$ nanoparticles. The positive curvature toward the rear corner ($T=50, \phi =3.0$) indicates that thermal enhancement is nonlinear and amplified by temperature. The smoothness of the surfaces confirms that the Hybrid model (GAM+GBM) successfully filtered experimental noise while capturing the underlying physics.

While partial dependence profiles isolate individual variables, nanofluid thermodynamics is inherently multivariate. To interrogate the coupled influence of operating conditions, we generated 3D response manifolds (Fig. 9) depicting the joint effect of Temperature (*T*) and Volume Fraction ($\phi$) on thermal conductivity. Consistent with the 1D profile analysis, these surfaces were constructed using Aluminum Oxide (Al$_2$O$_3$) as the reference nanoparticle to isolate the fluid-dynamic and thermal synergies from categorical solid-state lattice effects.

These surfaces reveal a distinct synergistic interaction mechanism consistent across all three base fluids:

1. *Thermal-Kinetic Coupling* The response surfaces are not planar; they exhibit a positive curvature toward the high-energy coordinate ($T=50^\circ$C, $\phi =3.0\%$). This non-additive behavior indicates that temperature acts as a catalyst for particle-driven enhancement. Physically, elevated temperatures reduce the base fluid’s kinematic viscosity, thereby intensifying the Brownian motion of the high-concentration nanoparticles. The Hybrid model successfully captures this physics: the marginal gain from adding nanoparticles is significantly higher at $50^\circ$C than at $20^\circ$C.
2. *Fluid-Specific Magnitude Scaling* While the topology of the interaction manifold remains invariant (confirming a universal enhancement mechanism), the absolute magnitude is strictly governed by the base fluid’s intrinsic properties:
  *Water (Fig.* 9*a)* Exhibits the steepest thermal gradient, peaking at $\approx 0.70$ W/mK. Water’s low viscosity facilitates maximum particle mobility and convective heat transfer.
  *Ethylene Glycol (Fig.* 9*b)* Shows a dampened response ($\approx 0.29$ W/mK), consistent with its higher viscosity which suppresses micro-convection currents.
  *Transformer Oil (Fig.* 9*c)* Displays the lowest conductivity ($\approx 0.127$ W/mK), dominated by the insulating nature of the oil matrix despite the nanoparticle loading.
3. *Topological Continuity (The GAM Advantage)* Crucially, the interaction manifolds are smooth and monotonic. This is a direct consequence of the Physics-Guided architecture: the GAM’s spline basis enforces differentiability across the domain. In contrast, pure “black-box” decision tree surfaces typically resemble jagged staircases. By constraining the manifold to be smooth, the Hybrid model ensures that the predicted thermodynamic landscape remains physically plausible, a mandatory requirement for stable engineering design and optimization.

### Statistical validity of residuals

**Fig. 10:**
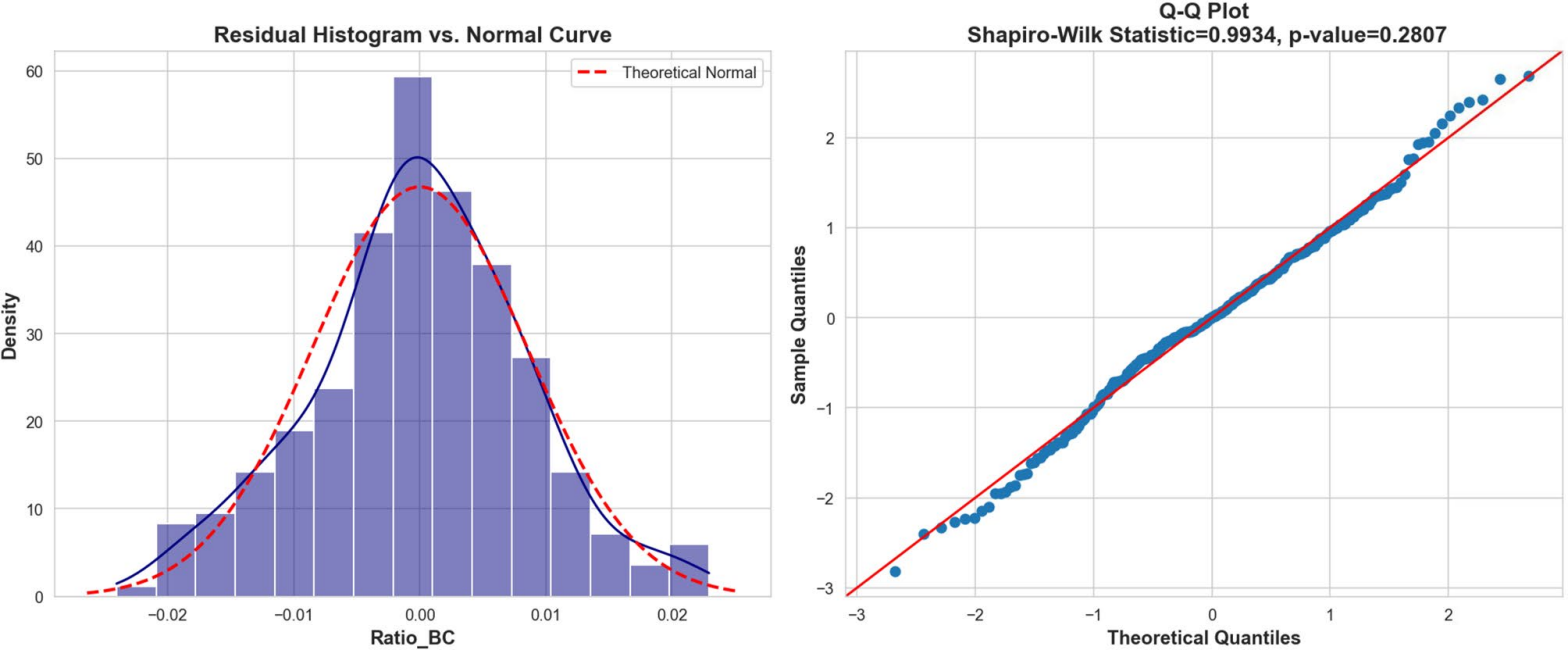
Statistical validation of model residuals. (Left) The histogram of Box-Cox transformed residuals shows a near-perfect Gaussian distribution. (Right) The Q-Q plot confirms linearity along the diagonal, and the Shapiro-Wilk test ($p=0.2807$) confirms normality, verifying that no systematic physical information remains in the error term.

Before establishing confidence intervals, it is imperative to verify that the model’s residuals contain no systematic bias. We examined the distribution of the Box-Cox transformed residuals, as visualized in Fig. 10.

The histogram (Left panel) exhibits a symmetric, bell-shaped distribution that aligns closely with the theoretical normal curve. This visual assessment is statistically corroborated by the Q-Q Plot (Right panel) and the Shapiro-Wilk normality test. The test yielded a statistic of $W=0.9934$ with a *p*-value of 0.2807. Since $p > 0.05$, we fail to reject the null hypothesis, effectively proving that the residuals are normally distributed. This Gaussian behavior is a robust indicator that the Hybrid model has fully resolved the deterministic physical trends, ensuring that the remaining error is purely stochastic.

### Uncertainty quantification and reliability analysis

Having validated the stochastic nature of the residuals, we proceeded to quantify the predictive confidence, which is essential for establishing safety margins in thermal management applications. Experimental measurements of nanofluid thermal conductivity are inherently susceptible to aleatoric (statistical) noise stemming from several distinct physical sources. These include calibration variations in the standard Transient Hot-Wire (THW) measurement technique, undocumented nanoparticle agglomeration over time (which inflates effective hydrodynamic size), and minor impurities within the base fluid chemistry.

Rather than yielding rigid point estimates that overfit to these measurement errors, the Hybrid architecture explicitly computes 95% PI to transparently quantify this underlying dataset uncertainty across the entire experimental domain.

**Fig. 11:**
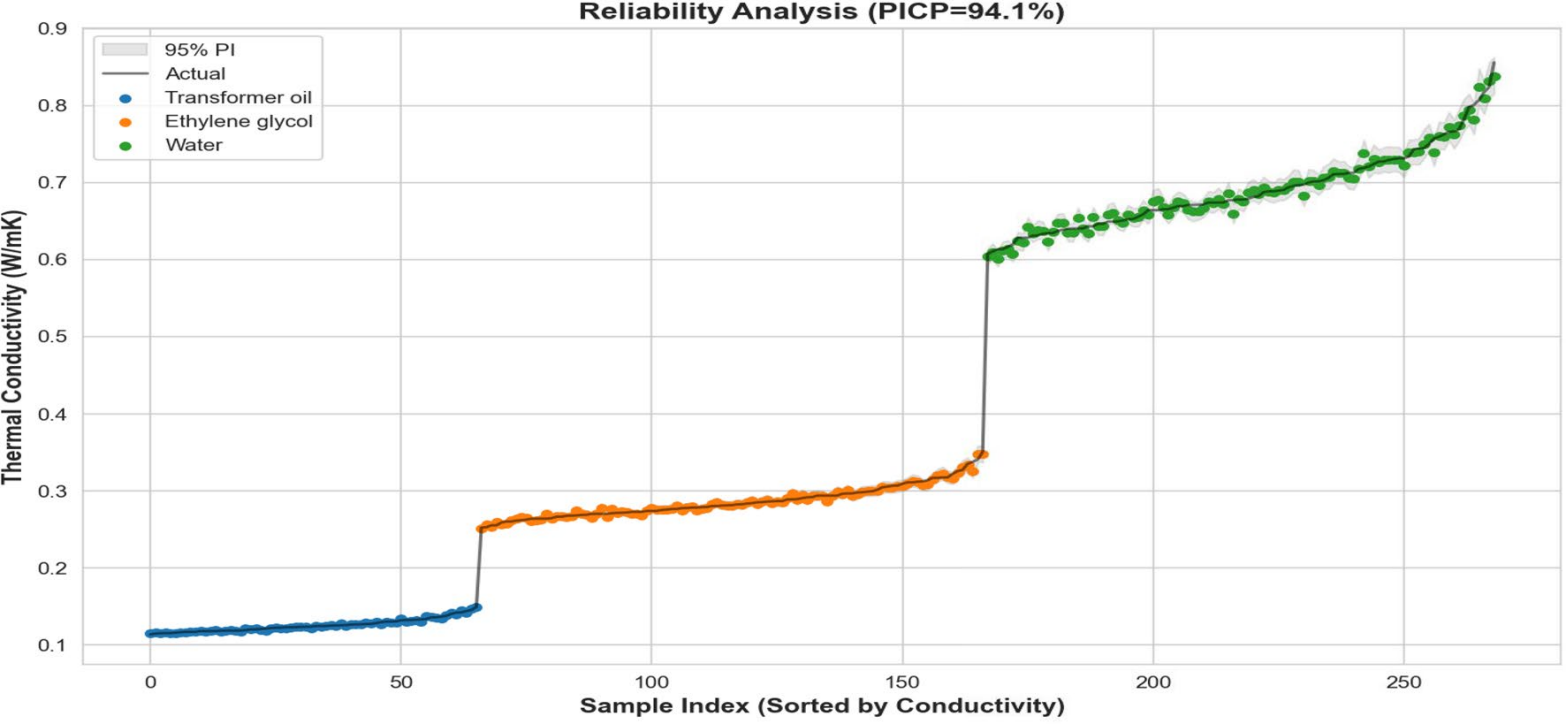
Reliability Analysis showing 95% Prediction Intervals (Grey Band) versus Actual Data (Solid Line). The model achieves a Coverage Probability (PICP) of 94.1%. The distinct “steps” correspond to Transformer Oil (Blue), Ethylene Glycol (Orange), and Water (Green), demonstrating that the model correctly scales its uncertainty estimates according to the physical stability of each base fluid.

Figure 11 visualizes these confidence bounds against the sorted experimental data, color-coded by base fluid type. The analysis yields a Prediction Interval Coverage Probability (PICP) of 94.1%, closely matching the nominal 95% target and confirming that the computed intervals are neither overly conservative nor excessively narrow for this dataset.

Key observations from the reliability analysis include:

1. *Fluid-Specific Uncertainty Regimes* The sorted index reveals three distinct confidence tiers corresponding to the base fluid properties:
  *Transformer Oil (Blue, Left)* Exhibits the narrowest prediction bands, indicating high model certainty in the low-conductivity, high-viscosity regime where particle motion and convective artifacts are severely restricted.
  *Ethylene Glycol (Orange, Center)* Shows intermediate variance, reflecting the transitional nature of its transport properties.
  *Water (Green, Right)* Displays the widest uncertainty intervals. This is physically consistent with the stochastic nature of aqueous nanofluids, where high temperatures induce vigorous Brownian motion, rapid transient aggregation, and more severe THW measurement noise.
2. *Absence of Structural Bias* Crucially, the experimental data (solid line) meanders randomly around the prediction centroid without systematic drift. This further confirms the findings of the residual analysis: the Hybrid GAM+GBM model has successfully eliminated structural bias, leaving only the irreducible aleatoric measurement uncertainty captured within the designated bounds.

### Generalization capability: leave-one-group-out (LOGO) analysis

While standard cross-validation assesses interpolation within the training distribution, the LOGO protocol evaluates the model’s ability to extrapolate to entirely unseen base fluids. This is the ultimate test of physical learning: can the model predict the behavior of a new fluid (e.g., Water) having never seen it during training?

**Fig. 12:**
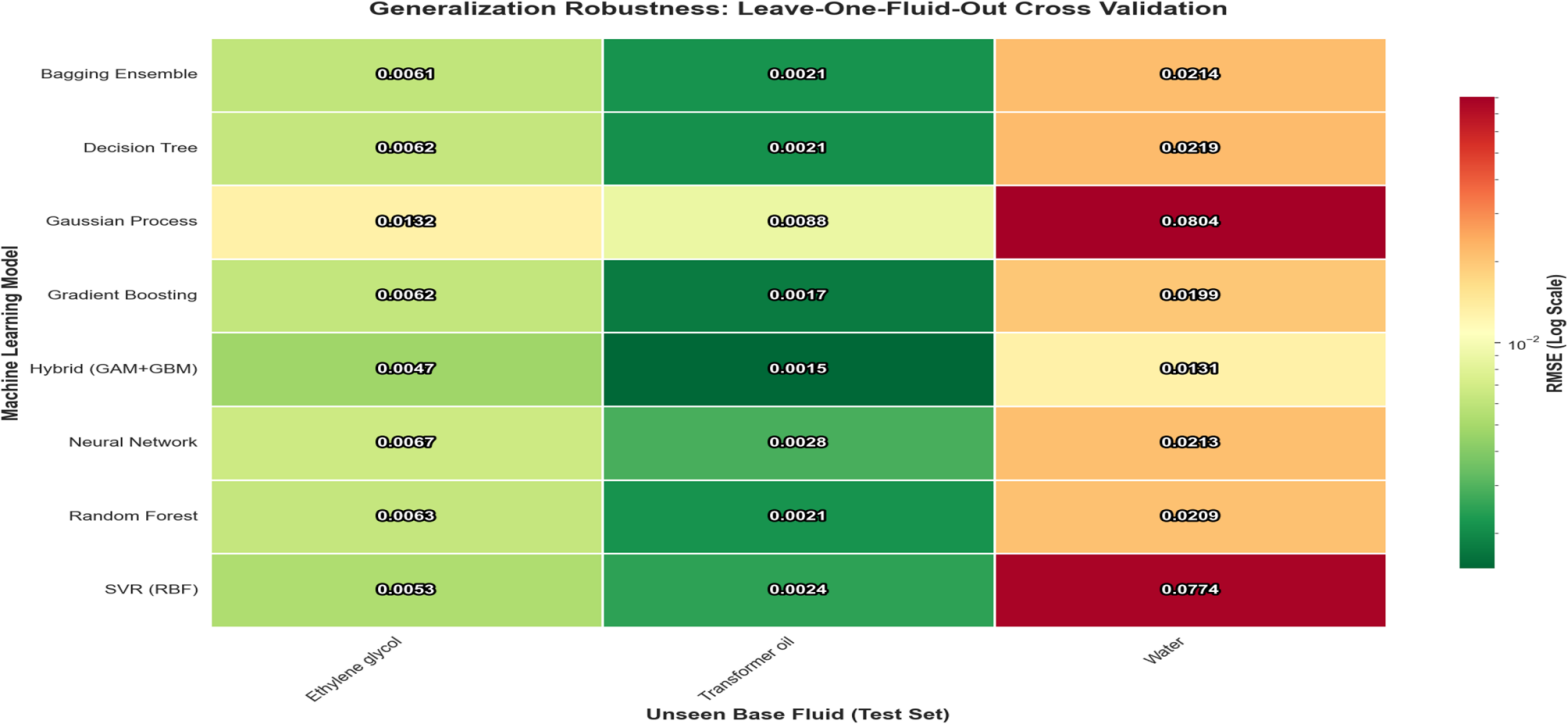
Generalization Robustness Heatmap. Values represent LOGO RMSE (W/mK) when the specified Base Fluid is excluded from training. The Hybrid (GAM+GBM) model dominates every scenario (greenest cells), particularly in the challenging “Water” extrapolation case where it outperforms the GP and SVR by a wide margin.

The results, summarized in the global ranking and visualized in Fig. 12, reveal a profound divergence in model capabilities.

#### Error distribution and stability

**Fig. 13:**
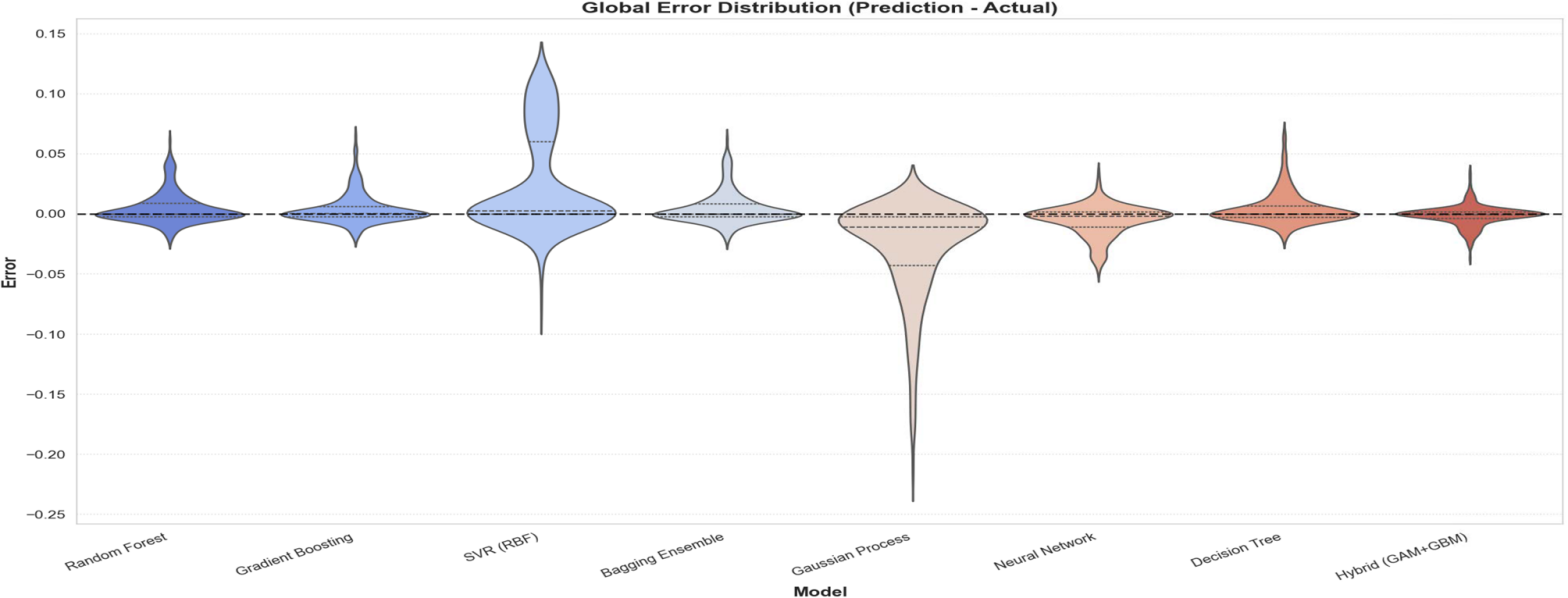
Global Error Distribution for LOGO cross-validation.

The structural stability of the model predictions is further analyzed in Fig. 13. The GP baseline exhibits a massive negative vertical tail, indicating a severe under-prediction bias when forced to extrapolate into unseen feature spaces during the LOGO cross-validation. Similarly, the SVR baseline suffers from an elongated positive tail, reflecting a pronounced tendency to over-predict under the same validation conditions. Conversely, the Hybrid model maintains the most compact, zero-centered error distribution. By combining the foundational thermodynamic trends of the Base Model with the precision of the residual learner (GBM), the Hybrid architecture effectively eliminates the extreme structural biases seen in both kernel-based methods while significantly narrowing the overall variance, confirming its suitability for robust engineering design.

#### Practical engineering implications and safety margins

While the LOGO cross-validation reveals an expected quantitative degradation when transitioning from interpolation (Standard CV) to extreme extrapolation (e.g., predicting water-based nanofluids), it is crucial to contextualize this apparent loss of fidelity within practical engineering tolerances. For the water exclusion scenario, the Hybrid model’s RMSE increases to 0.0131 W/mK. Given that the mean thermal conductivity of the water-based nanofluid samples in our dataset is  0.6897  W/mK, this absolute error translates to a relative deviation of approximately 1.9%.

In standard thermal engineering practice, the inherent experimental uncertainty of commercial thermal conductivity measurement devices (such as transient hot-wire analyzers) typically ranges from $\pm 3\%$ to $\pm 5\%$ ^32,33^. Therefore, even under the most severe extrapolation conditions tested, the Hybrid model’s predictive error as quantified by RMSE remains safely within the experimental noise floor of physical laboratory measurements. This mathematically confirms that the model’s extrapolative degradation does not compromise practical safety margins, rendering the hybrid architecture highly reliable for real-world thermal system design and nanofluid formulation.

#### Global performance hierarchy

The Hybrid (Base+GBM) model achieved a global LOGO mean RMSE of 0.0064 W/mK, establishing a decisive lead over all purely data-driven architectures in the LOGO cross-validation framework.

- *Superior Generalization* The Hybrid model outperformed the nearest purely data-driven competitor (GBM, LOGO mean RMSE 0.0092) by approximately 30.4%, proving the value of the physics-guided foundational splines.
- *Resilience of Boosting* The standalone GBM model secured second place among the black-box architectures, demonstrating that tree-based ensemble methods can generalize reasonably well if hyperparameters are tuned conservatively. However, it still lags significantly behind the hybrid baseline.
- *Failure of Kernel Methods* The GP exhibited catastrophic failure in extrapolation, yielding a LOGO mean RMSE of 0.0341 more than five times higher than the Hybrid model. This highlights a critical limitation of stationary kernels when predicting outside the specific feature space on which they were calibrated.

#### Scenario-specific robustness

Figure 12 breaks down the error metrics by the specific fluid excluded from training. This heatmap elucidates the mechanism of the Hybrid model’s success:

1. *The “Water” Challenge (High-Conductivity Extrapolation)* Predicting Water (Right column) is the most difficult task because its thermal conductivity ($\sim 0.6$ W/mK) is significantly higher than the training fluids (Oil and Glycol, $<0.3$ W/mK). Standard models struggled severely to extrapolate this trend, with SVR and GP yielding massive RMSEs of 0.0774 and 0.0804, respectively. In stark contrast, the Hybrid model achieved an RMSE of 0.0131, significantly mitigating the extrapolation error. By relying on the spline-based physics of the “Base Conductivity Proxy,” the Hybrid model correctly inferred that a higher fluid baseline mandates a steeper thermal enhancement curve.
2. *The “Oil” Baseline (Low-Conductivity Interpolation)* Predicting Transformer Oil (Center column) represents a relatively easier interpolation task, as its conductivity is bracketed by Water and Ethylene Glycol. Here, the Hybrid model achieved near-perfect precision (RMSE 0.0015), demonstrating that the architecture does not sacrifice local interpolation accuracy in its pursuit of global extrapolation robustness.

In summary, the LOGO analysis proves that the Physics-Guided Hybrid framework does not merely memorize data patterns; it successfully encodes the underlying physical laws of nanofluid heat transfer, allowing it to adapt to novel fluids with a level of reliability that opaque, black-box models cannot match.

#### Limitations and boundaries of extrapolation

While the LOGO validation protocol demonstrated the framework’s robust ability to extrapolate to unseen base fluids via the continuous thermodynamic proxy ($\tilde{k}_{base}$), it is critical to explicitly define the physical boundaries governing this generalization. The predictive reliability of the proposed PGML architecture is strictly bounded by the underlying heat transfer mechanisms active within the training domain.

Consequently, extrapolation is expected to fail or degrade under the following out-of-domain conditions:

- *Non-Newtonian and Liquid Metal Fluids* The model was trained on conventional Newtonian base fluids where thermal transport is governed by molecular collisions and intermolecular diffusion. Applying the framework to liquid metals, where free-electron transport dominates and conductivity values exceed those of the training fluids by an order of magnitude, would violate the thermodynamic range of the learned splines. Similarly, complex non-Newtonian fluids, whose shear-dependent viscosity fundamentally alters Brownian micro-convection, represent a mechanistic regime outside the model’s calibration domain.
- *Phase-Change and Supercritical Regimes* The GAM baseline assumes a continuous, single-phase liquid state. Extrapolating to temperatures approaching the boiling point, where localized nucleation or phase-change heat transfer initiates, will render the current baseline invalid.
- *Macroscopic and Slurry Geometries* The framework successfully captures the ”anomalous enhancement” driven by the interfacial nanolayer and Brownian motion. However, if extrapolated to micro-scale particles ($S > 1000$ nm) where Brownian motion ceases, or to extreme volume fractions ($\phi > 10\%$) where the suspension behaves as a dense slurry rather than a dispersed fluid, the effective medium dynamics will shift drastically, exceeding the calibration range of the model.

Acknowledging these boundaries ensures the framework is deployed safely within relevant thermophysical applications, while highlighting necessary pathways for future model expansion.

### Engineering applications in real-time design and optimization

The millisecond-scale inference latency of the Hybrid model, requiring no GPU acceleration, enables direct embedding into real-time thermal control loops. In applications such as smart HVAC systems^34^ or transformer monitoring^35^, the model can instantaneously predict conductivity under dynamic operating conditions, allowing control systems to adjust flow rates before critical thresholds are reached.

Beyond real-time control, the smooth and differentiable nature of the GAM baseline makes the framework a physically consistent surrogate for system-level optimization. By coupling the Hybrid model with metaheuristic algorithms such as Genetic Algorithms or Particle Swarm Optimization^36^, engineers can efficiently explore the thermodynamic design space, identifying the optimal combination of nanoparticle material, size, and volume fraction for a target conductivity without resorting to exhaustive Computational Fluid Dynamics (CFD) simulations or iterative laboratory testing^37,38^. Crucially, the LOGO validation results provide confidence that such optimization remains reliable even when the target operating condition involves a base fluid or concentration regime not explicitly present in the training data.

## Conclusion

This study addressed the fundamental challenge in thermophysical modeling of nanofluids, namely achieving a balance between predictive accuracy and physical interpretability. To this end, a physics-guided hybrid framework was developed by integrating a Generalized Additive Model (GAM) with a Gradient Boosting Machine (GBM) for predicting the thermal conductivity of metal-oxide nanofluids. The proposed framework decouples the learning process into two complementary components: a physics-consistent global trend captured by the GAM using spline-based representations, and a data-driven local correction modeled through a regularized boosting scheme. This structure enables the model to retain interpretability while effectively capturing complex nonlinear interactions present in experimental data. The results demonstrate that the hybrid model achieves high predictive accuracy (RMSE $\thickapprox 0.0048$ W/mK), comparable to state-of-the-art ensemble methods, while maintaining physically meaningful behavior. The analysis of partial dependencies confirms that the model successfully captures the expected thermodynamic trends with respect to temperature, particle volume fraction, and particle size. Furthermore, the generalization capability of the framework was evaluated using the Leave-One-Group-Out (LOGO) protocol. The hybrid model exhibited stable performance when extrapolated to unseen base fluids, indicating its ability to learn transferable thermophysical patterns rather than relying solely on data memorization. In addition, the model demonstrated consistent and smooth response surfaces, avoiding the discontinuities typically associated with tree-based models. The uncertainty analysis, supported by a Prediction Interval Coverage Probability (PICP) of approximately $94.1\%$, further confirms the reliability of the predictions within the investigated domain. From a practical perspective, conventional machine learning models such as Gradient Boosting remain effective for interpolation tasks within well-characterized datasets. However, for applications requiring improved interpretability, physical consistency, and generalization to new operating conditions, the proposed hybrid framework offers a promising and balanced modeling alternative. Overall, this study highlights the potential of physics-guided hybrid modeling strategies as a robust approach for thermophysical property prediction, bridging the gap between purely data-driven models and physically interpretable formulations.

### Future research directions

While the proposed Hybrid Physics-Guided Machine Learning (PGML) framework demonstrates high efficacy in predicting thermal conductivity, future research must prioritize extending this architecture to other critical thermophysical properties, such as dynamic viscosity, specific heat capacity, and density, to provide comprehensive nanofluid characterization. Furthermore, a highly promising frontier for future work involves the transition from structurally constrained machine learning to true Physics-Informed Neural Networks (PINNs) ^39^. While the current GAM-based approach successfully enforces physical constraints via continuous splines, PINNs offer the advanced capability to embed actual physical governing equations, such as the Navier-Stokes and energy conservation equations, directly into the loss function of the neural network. From a computational solver perspective, the feasibility of coupling these PINNs with localized property predictors is immense. Instead of relying purely on static regression, future architectures could act as surrogate solvers, simultaneously calculating the dynamic physical governing equations and the localized thermophysical properties. This deep integration would enable real-time, high-fidelity simulations of complex nanofluidic heat transfer systems without the severe computational bottlenecks associated with traditional, iterative Computational Fluid Dynamics (CFD) finite-element solvers.

In parallel with advanced solver integration, future investigations should incorporate rigorous global sensitivity analyses (e.g., variance-based Sobol indices or Shapley Additive Explanations). While the partial dependence profiles presented in Section Interpretation of physical dependencies and data sparsity successfully isolate the marginal thermodynamic behaviors of individual inputs, formal global sensitivity frameworks would provide a definitive quantitative ranking of feature importance. By mathematically decoupling the interacting influences of nanoparticle concentration, base fluid rheology, and operating temperature, researchers can determine which parameters dominate thermal transport under specific conditions. As demonstrated in recent computational mechanics and systems engineering literature^40–43^, integrating quantitative sensitivity analyses significantly enhances model interpretability. In the context of nanofluids, the primary contribution of this approach would be to streamline real-world thermal system design, allowing engineers to prioritize the most impactful control variables for targeted property optimization rather than relying on costly trial-and-error experimentation.

## Data Availability

The data supporting the findings of this study were derived from previously published research. All data analyzed during this study are available within the cited source and are referenced accordingly in this manuscript.

## Abbreviations

AIC: Akaike information criteria
BIC: Bayesian information criteria
BS: B-Spline basis functions
CFD: Computational fluid dynamics
CI: Confidence interval
CV: Cross-validation
DT: Decision trees
GAM: Generalized additive model
GBM: Gradient boosting machine
GP: Gaussian process
LOGO: Leave-one-group-out (generalization protocol)
MAE: Mean absolute error
MLP: Multilayer perceptron (neural network)
PGML: Physics-guided machine learning
PI: Prediction interval
PICP: Prediction interval coverage probability
PINN: Physics-informed neural network
RBF: Radial basis function (kernel)
ReLU: Rectified linear unit (activation function)
RF: Random forest
RMSE: Root mean squared error
SVR: Support vector regression
SHAP: Shapley additive explanations

## List of symbols

*C*: Regularization parameter (SVR cost function)
*d*: Maximum tree depth (GBM structural constraint)
$h_m$: Weak learner function (boosting tree)
$\mathbb {I}(\cdot )$: Binary indicator function (categorical fixed effects)
$k_{nf}$: Thermal conductivity of nanofluid [W/m$\cdot$K]
$k_{base}$: Thermal conductivity of base fluid [W/m$\cdot$K]
$k_{ratio}$: Conductivity enhancement ratio ($k_{nf}/k_{base}$)
$\tilde{k}_{base}$: The normalized base fluid conductivity (physics proxy)
$k_{Leaf}$: Minimum samples per leaf node (regularization constraint)
$k_{min}$: Minimum samples required to split an internal node
*M*: Number of estimators (trees) in the ensemble
$y^{(\lambda )}$: Box-Cox transformed conductivity ratio
$\alpha$: Regularization term (MLP) / kernel parameter (GP)
$\gamma$: Kernel coefficient (RBF/SVR)
$\epsilon$: Epsilon-tube margin (SVR loss function)
$\eta$: Stochastic subsampling fraction (GBM)
$\lambda$: Box-Cox transformation parameter
$\nu$: Learning rate (gradient boosting shrinkage)
$\phi$: Particle volume fraction [%]
$\Omega$: Set of structural regularization constraints
*T*: Temperature [$$^\circ$$C]
$$T_c$$: Centered temperature ($$T - \bar{T}$$)
*S*: Particle size [nm]
*R*: Residuals ($$y_{actual} - y_{GAM}$$)
$$\sigma$$: Standard deviation
$\mu _{train}$: Mean of base fluid conductivity (training set only)
$\sigma _{train}$: Standard deviation of base fluid conductivity (training set only)

## References

[1] Stephen, C. U. S. Enhancing thermal conductivity of fluids with nanoparticles. In* ASME international Mechanical Engineering Congress and Exposition*, vol. 17421, pp. 99–105. American Society of Mechanical Engineers, (1995).

[2] Clerk, M. J. *A treatise on electricity and magnetism* Vol. 1 (Clarendon press, 1873).

[3] Lee Hamilton, R. & Crosser, Orrin K. Thermal conductivity of heterogeneous two-component systems. *Ind Eng Chem Fundam* **1**(3), 187–191 (1962).

[4] Das, Sarit Kumar, Putra, Nandy Setiadi Djaya., Thiesen, Peter & Roetzel, Wilfried. Temperature dependence of thermal conductivity enhancement for nanofluids. *J Heat Transfer* **125**(4), 567–574 (2003).

[5] Hojjat, M., Gh Etemad, S., Bagheri, R. & Thibault, J. Thermal conductivity of non-newtonian nanofluids: Experimental data and modeling using neural network. *Int J Heat Mass Transfer* **54**(5–6), 1017–1023 (2011).

[6] Vajjha, Ravikanth S. & Das, Debendra K. Experimental determination of thermal conductivity of three nanofluids and development of new correlations. *Int J Heat Mass Transfer* **52**(21–22), 4675–4682 (2009).

[7] Tajik Jamal-Abadi, M. & Zamzamian, A. H. Optimization of thermal conductivity of al2o3 nanofluid by using ANN and GRG methods. *Int J Nanosci Nanotechnol* **9**(4), 177–184 (2013).

[8] Battira, M. & Bessaïh, Rachid. Radial and axial magnetic fields effects on natural convection in a nanofluid-filled vertical cylinder. *J Appl Fluid Mech* **9**(1), 407–418 (2015).

[9] Noghrehabadi, Aminreza & Pourrajab, Rashid. Experimental investigation of forced convective heat transfer enhancement of -al2o3/water nanofluid in a tube. *J Mech Sci Technol* **30**(2), 943–952 (2016).

[10] Beck, M. P., Yuan, Y., Warrier, P. & Teja, A. S. The effect of particle size on the thermal conductivity of alumina nanofluids. *J Nanoparticle Res* **11**(5), 1129–1136 (2009).

[11] Murshed, S. M. S., Leong, K. C. & Yang, C. Thermophysical and electrokinetic properties of nanofluids: a critical review. *Appl Thermal Eng* **28**(17–18), 2109–2125 (2008).

[12] Tahani, M., Vakili, M. & Khosrojerdi, S. Experimental evaluation and ANN modeling of thermal conductivity of graphene oxide nanoplatelets/deionized water nanofluid. *Int Commun Heat Mass Transfer* **76**, 358–365 (2016).

[13] Longo, G. A., Zilio, C., Ceseracciu, E. & Reggiani, M. Application of artificial neural network (ANN) for the prediction of thermal conductivity of oxide-water nanofluids. *Nano Energy* **1**(2), 290–296 (2012).

[14] Bhaumik, Bivas, Changdar, Satyasaran & De, Soumen. An expert model based on physics-aware neural network for the prediction of thermal conductivity of nanofluids. *J Heat Transfer* **144**(10), 103501 (2022).

[15] Alade, I. O. et al. Modeling thermal conductivity enhancement of metal and metallic oxide nanofluids using support vector regression. *Adv Powder Technol* **29**(1), 157–167 (2018).

[16] Rasmussen, C.E. Gaussian processes in machine learning. In Summer school on machine learning, pp. 63–71. Springer, (2003).

[17] Rajakumar, M. P. et al. Performance enhancement of photovoltaic thermal collectors using water based mno2 nanofluids and machine learning models. *Sci Rep* **15**(1), 39826 (2025). 41233458 10.1038/s41598-025-23505-x PMC12615616

[18] Kanti, Praveen Kumar, Prabhakar Sharma, V., Wanatasanappan, Vicki & Said, Nejla Mahjoub. Explainable machine learning techniques for hybrid nanofluids transport characteristics: An evaluation of shapley additive and local interpretable model-agnostic explanations. *J Thermal Anal Calorim* **149**(21), 11599–11618 (2024).

[19] Zhang, Xi. et al. A prediction model for dyke-dam piping based on data augmentation and interpretable ensemble learning. *Eng Fail Anal* **142**, 110174 (2025).

[20] Liu, Bokai et al. Explainable machine learning for multiscale thermal conductivity modeling in polymer nanocomposites with uncertainty quantification. *Compos Struct* **370**, 119292 (2025).

[21] Liu, Bokai et al. Al-demat: A web-based expert system platform for computationally expensive models in materials design. *Adv Eng Softw* **176**, 103398 (2023).

[22] Liu, Bokai & Weizhuo, Lu. Surrogate models in machine learning for computational stochastic multi-scale modelling in composite materials design. *Int J Hydromechatronics* **5**(4), 336–365 (2022).

[23] Liu, Bokai, Vu-Bac, Nam & Rabczuk, Timon. A stochastic multiscale method for the prediction of the thermal conductivity of polymer nanocomposites through hybrid machine learning algorithms. *Compos Struct* **273**, 114269 (2021).

[24] Liu, Bokai, Vu-Bac, Nam, Zhuang, Xiaoying, Xiaolong, Fu. & Rabczuk, Timon. Stochastic full-range multiscale modeling of thermal conductivity of polymeric carbon nanotubes composites: A machine learning approach. *Compos Struct* **289**, 115393 (2022).

[25] Liu, Bokai, Vu-Bac, Nam, Zhuang, Xiaoying, Xiaolong, Fu. & Rabczuk, Timon. Stochastic integrated machine learning based multiscale approach for the prediction of the thermal conductivity in carbon nanotube reinforced polymeric composites. *Compos Sci Technol* **224**, 109425 (2022).

[26] Liu, Bokai, Weizhuo, Lu., Olofsson, Thomas, Zhuang, Xiaoying & Rabczuk, Timon. Stochastic interpretable machine learning based multiscale modeling in thermal conductivity of polymeric graphene-enhanced composites. *Compos Struct* **327**, 117601 (2024).

[27] Xia, Yangyang et al. Prediction of bending strength of glass fiber reinforced methacrylate-based pipeline uv-cipp rehabilitation materials based on machine learning. *Tunn Undergr Space Technol* **140**, 105319 (2023).

[28] Wood, S.N., Goude, Y. & Fasiolo, M. Interpretability in generalized additive models. In interpretability for industry 4.0: statistical and machine learning approaches, pp. 85–123. Springer, (2022).

[29] Trevor, H., Robert, T. Generalized additive models, vol. 1. *Institute of Mathematical Statistics* (1986).

[30] Patel, H. E., Sundararajan, T. & Das, S. K. An experimental investigation into the thermal conductivity enhancement in oxide and metallic nanofluids. *J Nanoparticle Res* **12**(3), 1015–1031 (2010).

[31] Yu, W. & Choi, A. S. The role of interfacial layers in the enhanced thermal conductivity of nanofluids: A renovated Maxwell model. *J Nanoparticle Res* **5**, 167–171 (2003).

[32] Kostic, M., Simham, K.C. Computerized, transient hot-wire thermal conductivity (hwtc) apparatus for nanofluids. In Proceedings of the 6th WSEAS International Conference on HEAT and MASS TRANSFER (HMT’09), pp. 71–78, (2009).

[33] Prado, Jose I., Calviño, Uxía. & Lugo, Luis. Experimental methodology to determine thermal conductivity of nanofluids by using a commercial transient hot-wire device. *Appl Sci* **12**(1), 329 (2021).

[34] Zhuang, Dian et al. Data-driven predictive control for smart hvac system in iot-integrated buildings with time-series forecasting and reinforcement learning. *Appl Energy* **338**, 120936 (2023).

[35] Ramesh, Jayroop, Sakib Shahriar, A. R., Al-Ali, Ahmed Osman & Shaaban, Mostafa F. Machine learning approach for smart distribution transformers load monitoring and management system. *Energies* **15**(21), 7981 (2022).

[36] Hai, T. et al. Optimizing ternary hybrid nanofluids using neural networks, gene expression programming, and multi-objective particle swarm optimization: A computational intelligence strategy. *Sci Rep* **15**(1), 1986 (2025). 39814861 10.1038/s41598-025-85236-3 PMC11736119

[37] Sabeur, A. Machine learning applications in nanofluid research: A comprehensive review of trends, challenges, and future perspectives. IntechOpen (2026).

[38] Mohan Rao, U., Issouf Fofana, T., Jaya, Esperanza Mariela, Rodriguez-Celis, Jocelyn Jalbert & Picher, Patrick. Alternative dielectric fluids for transformer insulation system: Progress, challenges, and future prospects. *IEEE Access* **7**, 184552–184571 (2019).

[39] Liu, Bokai, Wang, Yizheng, Rabczuk, Timon, Olofsson, Thomas & Weizhuo, Lu. Multi-scale modeling in thermal conductivity of polyurethane incorporated with phase change materials using physics-informed neural networks. *Renew Energy* **220**, 119565 (2024).

[40] Liu, Bokai, Liu, Pengju, Weizhuo, Lu. & Olofsson, Thomas. Explainable artificial intelligence (xai) for material design and engineering applications: A quantitative computational framework. *Int J Mech Syst Dyn* **5**(2), 236–265 (2025).

[41] Zhang, Xi. et al. An archimedes optimization algorithm based extreme gradient boosting model for predicting the bending strength of uv cured glass fiber reinforced polymer composites. *Polymer Compos* **47**(5), 4228–4245 (2026).

[42] Liu, Bokai, Vu-Bac, Nam, Zhuang, Xiaoying & Rabczuk, Timon. Stochastic multiscale modeling of heat conductivity of polymeric clay nanocomposites. *Mech Mater* **142**, 103280 (2020).

[43] Liu, Bokai et al. Data-driven quantitative analysis of an integrated open digital ecosystems platform for user-centric energy retrofits: A case study in northern Sweden. *Technol Soc* **75**, 102347 (2023).

